# Stress-testing the EU energy system: Modeling resilience without Russian gas

**DOI:** 10.1016/j.isci.2026.115866

**Published:** 2026-06-01

**Authors:** Chi Kong Chyong, Henrik Schmidt

**Affiliations:** 1 Oxford Institute for Energy Studies, Oxford, England; 2 Oxford Institute for Energy Studies & School of Public Affairs, Sciences Po Paris, Paris, France

**Keywords:** Energy sustainability, Energy systems, Energy modeling, Economics

## Abstract

Europe’s gas and electricity markets are deeply intertwined, making the system vulnerable to compound supply and demand shocks—particularly following the loss of Russian pipeline gas. Using a global coupled gas-electricity partial-equilibrium model that incorporates LNG trade, storage behavior, demand-side response, and multiple weather scenarios, we show that Europe can generally maintain physical supply even under severe stress. The principal vulnerability lies not in outright shortages but in sharp, asymmetric price spikes, especially during cold winters or when LNG supply is constrained. LNG import capacity, gas storage, hydropower, and fuel switching in the power sector act as key stabilizers, though network bottlenecks persist in Eastern and Southern Europe. Accelerated renewables deployment materially reduces exposure to gas-linked price volatility, whereas additional Russian LNG has only a marginal impact on prices. These findings point to the importance of system-wide stress testing, targeted infrastructure investment, and reducing gas’s role as the marginal price setter in electricity markets.

## Highlights

• Accelerated renewables cut EU gas prices by 29% and power prices by 46%
• Banning Russian LNG has limited effects on wholesale prices
• Reintroducing Russian pipeline gas lowers prices by up to 23% but benefits fade by 2031
• EU energy resilience hinges on weather, LNG shocks, and renewable deployment

## Graphical abstract

**Figure:**
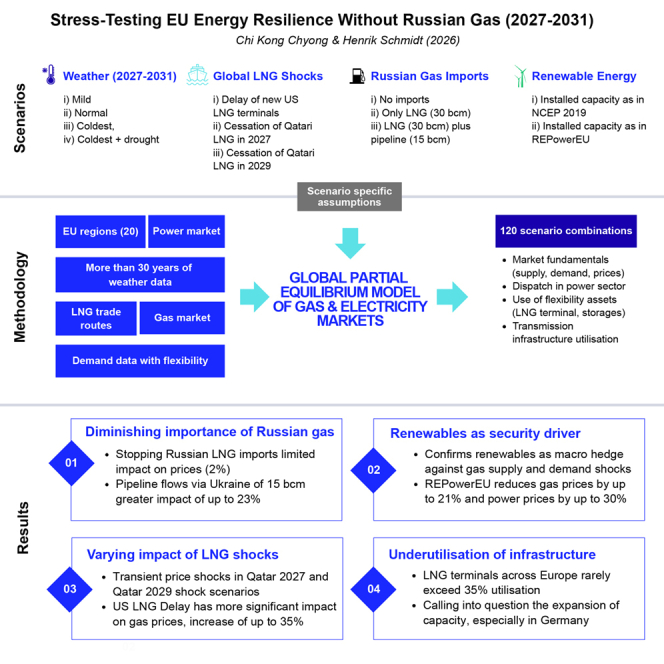

## Introduction

Natural gas is an important energy source for European economies, representing roughly a quarter of total primary energy consumption^1^ and more than one-fifth of final consumption.^2^ In residential (RES) energy consumption, natural gas represents one-third of all energy consumption and is mostly used for space and water heating and cooking.^2^ Natural gas is also a key fuel in the electricity sector, providing 20% of the energy power plants require to generate electricity and heat. Lastly, in the industrial (IND) sector, natural gas accounts for at least one-third of energy consumption, the highest share among competing energy sources.^3^ Historically, Russia was the largest gas supplier to the European Union (EU), providing in 2021 ca. about 40% of all European gas import needs.^4^ On February 24, 2022, Russia launched a full-scale military invasion of Ukraine, leading the European Commission (EC) to develop plans to eliminate dependence on Russian natural gas by the end of 2027.^5^ At the same time, in 2024, EU Member States (MS) imported record amounts of Russian Liquified Natural Gas (LNG), with the inflow rising from 13 bcm in 2021 to 21 bcm in 2024.^6^ Still, Europe lost roughly two-thirds of its natural gas supplies from Russia in recent years, falling from 157 bcm in 2021 to 54 bcm in 2024.^6^ A “bridge” no longer exists for the transcontinental natural gas trade between Russia and Europe.

In May 2025, the EC reiterated its goal of stopping all remaining Russian energy imports by the end of 2027.^7^ A consecutive legislative proposal was presented in June 2025 to lay the legal groundwork,^8^ and Members of the European Parliament voted by large margins in favor of the legislation in October 2025.^9^ Consequently, a complete cessation of Russian natural gas deliveries to Europe has turned from a theoretical and market modeling exercise (on energy modeling and simulation studies concerning quantification of impacts of loss of Russian energy see, e.g., Monforti and Szikszai^10^; Lochner^11^; Szikszai and Monforti^12^; Chyong and Hobbs^13^; Richter and Holz^14^; Egging and Holz^15^; Baltensperger et al.^16^; Bouwmeester and Oosterhaven^17^; Deane et al.^18^; Sesini et al.^19^; Sesini et al.^20^) to reality.

Conventionally, energy security is defined as “the uninterrupted availability of energy sources at an affordable price.”^21^ Closely linked to energy security is the concept of energy resilience, which focuses on an energy system’s ability to withstand and recover from stress events. Energy resilience can be defined as “the ability to withstand and reduce the magnitude and/or duration of disruptive events, which includes the capability to anticipate, absorb, adapt to, and/or rapidly recover from such an event.”^22^ In contrast, Roege et al.^23^ define energy resilience more simply as “the ability of a system to recover from adversity.” Building on this, Umunnakwe et al.^24^ introduced the “resilience trapezoid,” which captures the sequential phases of a system’s response to disruption: withstanding, absorbing, restoring, and adapting. These stages provide a useful analytical lens for interpreting the behavior of energy systems under high-impact, low-probability (HILP) events.

Historically, energy resilience has been assessed using infrastructure-based metrics, most notably the *N*-1 criterion, which tests whether a system can continue operating after losing a single component.^22^ As Dickel et al.^25^ write, “this standard requires MS to ensure that, in case of disruption of their single largest piece of gas infrastructure, the capacity of the remaining infrastructure could satisfy an exceptionally high demand level; it also requires developing physical reverse-flow capacity subject to a potential cost/benefit analysis.”

However, the 2021–2023 energy crisis exposed the limitations of such static approaches, as they failed to capture the interdependence of energy markets, behavioral responses, and supply demand dynamics. Following these events, the literature has moved toward broader frameworks that distinguish between attribute-based indicators, which describe static system properties such as generation diversity or network redundancy, and performance-based indicators, which measure how the system behaves under stress. Only a selection of the literature can be presented here. More information can be found in Roege et al.,^23^ Willis and Loa,^26^ Panteli et al.,^27^ and Vugrin et al.^28^

Martišauskas et al.^29^ for example, propose a framework combining both perspectives: attribute-based indicators such as the number of fuel entry points, generation diversity, and transmission capacity, and performance-based metrics such as unmet demand or changes in supply costs. Similarly, the Institute of Energy Economics (EWI)^30^ report on German gas supply resilience quantifies resilience using price and demand reactions, diversification of import sources, and utilization rates of key infrastructures, including LNG terminals and pipelines. The diversification of energy imports can serve as a metric to assess the security of an energy system against supply side shocks. Diversity indicators such as the Herfindahl-Hirschman index (HHI) or the Shannon-Wiener index (SWI) are applied in this regard.^29^ Other studies extend the concept to regulatory and environmental dimensions: Baldursson et al.^22^ discuss how resilience regulation in Europe must evolve to account for growing exposure to natural disasters, while Jasiūnas et al.^31^ qualitatively analyze multiple threat categories—including extreme temperatures, precipitation, wind speeds, and cyberattacks—affecting energy system resilience.

In this study, resilience is operationalized as a dynamic energy system property that emerges endogenously from the interaction of economic and technical processes within the model. Drawing on the resilience trapezoid, we interpret resilience through measurable performance-based indicators corresponding to the four response phases (Table 1). Economic responses capture the system’s ability to absorb and adapt through market mechanisms such as price movements and demand-side reactions. These include variations in wholesale gas and electricity prices and the magnitude of demand-side reductions in the IND and RES sectors. Technical-physical responses reflect the capacity to maintain and restore supply through operational flexibility and infrastructure utilization. These include hydro and underground gas storage, fuel switching in the power sector, LNG inflow adjustments, and network reconfiguration through pipelines and power transmission lines. Thus, these indicators capture how the system withstands stress, absorbs shocks through flexibility, restores normal operation after disruption, and adapts through behavioral and structural change.

**Table 1:** Operationalization of resilience indicators used in this study

Dimension	Indicator reported in results	Primary resilience stage(s)	Interpretations
Economic	Δ wholesale gas/electricity prices; volatility (implied by ranges); cross-MS price dispersion	absorbing, adapting	large/sustained spikes → weak absorption; narrowing dispersion → effective adaptation/integration
Economic	industrial/residential DSR volumes (bcm/TWh-th)	adapting (also absorbing)	higher DSR at lower price thresholds → stronger adaptive capacity
Technical-physical (operational)	gas and hydro storage levels; depth of drawdown; recovery trajectory	absorbing, restoring	deep winter drawdowns with timely summer refill → healthy absorb/restore cycle
Technical-physical (operational)	fuel switching in power (gas→coal/hydro; gas→RES)	withstanding, absorbing	rapid substitution at stress points without load shedding
Technical-physical (network)	pipeline and power interconnector utilization	withstanding, absorbing	high utilization without unmet demand → network supports shock absorption
Technical-physical (network)	LNG inflow ramping; imports terminal utilization	withstanding, absorbing	ability to ramp imports in stress years (e.g., coldest+) without bottlenecks

Notes: Indicators are computed endogenously by the model.

We interpret withstanding as maintaining supply without unmet demand; absorbing as limiting the magnitude and duration of price spikes while drawing on flexibility without breaching operational limits; restoring as timely storage refilling and re-convergence of prices and flows to pre-shock levels; and adapting as enduring behavioral or structural adjustments, such as sustained demand-side flexibility or reconfiguration of trade flows.

While these four stages can be associated with quantitative indicators, their interpretation remains context-dependent. Thresholds that delineate, for instance, an absorptive versus a restorative response depend not only on the magnitude of deviation from baseline conditions but also on its duration, frequency, and system-wide impact. A brief price spike may represent a normal market reaction if flexibility sources are rapidly mobilized, whereas a sustained price increase over a year could signal structural stress. Similarly, the acceptable depth of storage drawdown or level of infrastructure utilization varies with regional supply diversity, regulatory settings, and policy tolerance for risk. Consequently, resilience in this study is evaluated comparatively and qualitatively by examining relative deviations, recovery speeds, and behavioral adjustments across scenarios, rather than through fixed numerical thresholds. This approach reflects the temporal and structural complexity of energy-system responses to compound shocks and avoids imposing arbitrary judgments about acceptable performance levels.

As the 2021/23 energy crisis showed, Europe’s energy system faces significant challenges during the transition to decarbonization from HILP shocks, emphasizing the importance of resilience in supply disruptions and extreme weather events. This situation underscores the necessity for diversified sources of flexibility, including the European power sector, end-use consumer demand-side responses (DSRs), extensive use of gas and hydropower storage, and flexible LNG supplies from the global market. Considering the integration of Europe into global energy markets and the shift toward renewable energy and away from Russian gas imports, this paper aims to answer the following question: how resilient is the European energy system to compound supply and demand shocks, and what role can market design and policy tools play in mitigating these risks?

Using different weather scenarios, ranging from an extremely cold winter to summer droughts, we model how the European energy market absorbs both supply and demand without Russian gas imports. Furthermore, we conduct several sensitivity analyses that allow for (1) various global LNG supply shock scenarios, (2) a limited return of Russian natural gas to Europe, and (3) a higher renewable energy penetration as outlined in the REPowerEU plan. Scenario narratives and assumptions are outlined in detail in the STAR Methods. See Table 2 for an overview of modelled scenarios.

**Table 2:** Overview of scenario combination

	Mild Winter	Normal Winter	Coldest Winter	Coldest Winter + Drought
Baseline	Baseline and Mild	Baseline and Normal	Baseline and Coldest	Baseline and Coldest+
US New Volume Delay (LNG scenario)	US Delay and Mild	US Delay and Normal	US Delay and Coldest	US Delay and Coldest+
Qatar Shock 2027 (LNG scenario)	QA27 and Mild	QA27 and Normal	QA27 and Coldest	QA27 and Coldest+
Qatar Shock 2029 (LNG scenario)	QA29 and Mild	QA29 and Normal	QA29 and Coldest	QA29 and Coldest+
Russian LNG Imports (sensitivity)	RU LNG and Mild	RU LNG and Normal	RU LNG and Coldest	RU LNG and Coldest+
Russian All Imports (sensitivity)	RU All and Mild	RU All and Normal	RU All and Coldest	RU All and Coldest+
REPowerEU (sensitivity)	REPowerEU and Mild	REPowerEU and Normal	REPowerEU and Coldest	REPowerEU and Coldest+

The rest of this paper proceeds as follows. In results section, we present and discuss key findings of our modeling. The presentation begins with the evolution of natural gas demand and supply (see natural gas demand elasticity section), gas and electricity prices (see gas and electricity price reactions section), and the dispatch of flexibility sources (see behavior of flexibility sources section). In country-level energy resilience across EU MS section, we discuss country-level results and the diverging energy resilience of European regions. For details on the partial equilibrium global energy market model, see STAR Methods. Model inputs are reported in supplemental information, (SI 1 data inputs). The global energy market was specifically developed for this study and explicitly integrates LNG imports, DSRs to price increases, and weather- and demand-driven changes in the power sector. To conclude this article, we present policy implications in discussion section, and limitations of this study in limitations of the study section.

## Results

This section presents the modeling results. The analysis focuses on the following energy resilience indicators: demand-side reactions, the evolution of wholesale gas and electricity prices, changes in the capacity factors of flexibility sources (hydro, underground gas storage, and LNG terminals), and utilization rates of energy infrastructure. While most of the data are presented as European averages, we also present national deviations in gas and electricity prices. Additionally, the analysis of the utilization rate of energy infrastructures in country-level energy resilence across EU MS section is conducted on the national level. For every indicator, we present the baseline scenario results first to highlight differences across the four weather years modeled here. Afterward, we present the results of the LNG scenarios for a normal weather year to analyze how disruptions to the LNG market affect the indicators. Lastly, within each sub-section, we present results for the sensitivity scenarios. Key model output data are aggregated in the supplemental information (S1_data).

### Natural gas demand elasticity

#### Baseline

**Figure 1:**
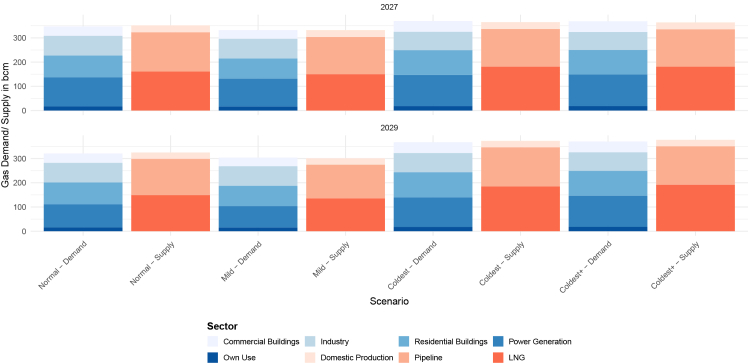
The EU27’s natural gas consumption and supply in the baseline scenario with different weather years The differences between total supply and total consumption in a given year arise from storage charging and discharging. Since the model assumes perfect foresight and optimizes over multiple years, gas can be stored in one year and withdrawn in another. As a result, annual supply and consumption may not be equal.

Figure 1 shows the evolution of natural gas demand and supply across the different weather scenarios in the baseline for two selected years, 2027 and 2029. Natural gas demand in the EU27 reaches 347 bcm in 2027 under a normal weather year, with the following sectoral structure: power generation (PWR, 120 bcm), followed by RES (90 bcm), industry (81 bcm), commercial (COM) buildings (39 bcm), and the energy industry’s own use (17 bcm). For reference, the EU’s natural gas demand fell from 390 bcm in 2021^32^ to 362 bcm in 2022 and 295 bcm in 2023.^33^ A recovery in gas demand from the IND and building sectors can explain the increase in natural gas demand between 2023 (295 bcm) and 2027 (347 bcm). Nevertheless, thanks to accelerating decarbonization, gas demand is set to fall from the reference year 2021 (390 bcm) by around 43 bcm, driven by lower demand in the power and buildings sectors.

In 2027, in a normal weather year, on the supply side, most natural gas arrives in Europe through pipeline imports (162 bcm) from Norway and Algeria, and via the UK. LNG shipments (161 bcm) provide the same supply, trailed by domestic production at 29 bcm. The latter is primarily due to gas production in the Netherlands, Romania, Germany, and Poland.

**Figure 2:**
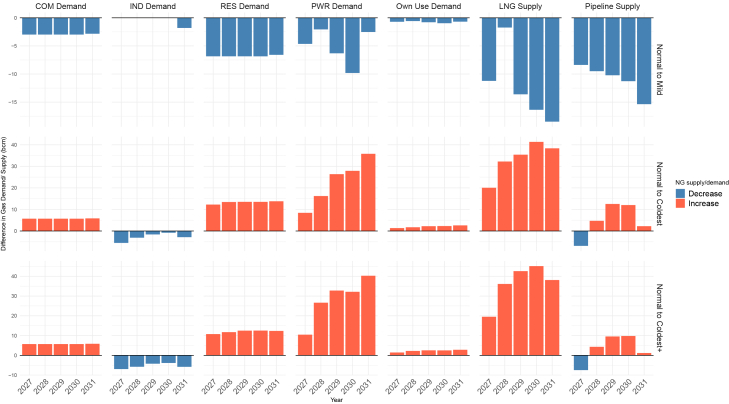
Evolution of gas supply and demand in the baseline scenarios, delta in bcm

**Figure 3:**
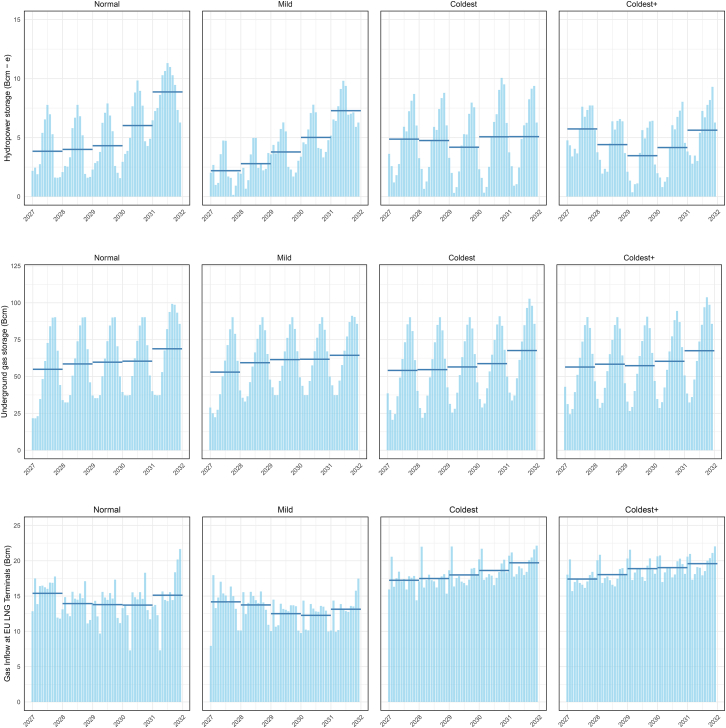
Use of flexibility sources in the weather scenarios The chart shows storage (large-scale gas and electricity storage) levels and changes in 2023–2031 (monthly level) from a baseline scenario to a scenario without access to Russian gas supplies and under a coldest+ scenario. The bars show monthly values, while the lines show the annual average.

Figure 2 provides a detailed breakdown of natural gas consumption and supply changes across the different weather scenarios in the baseline. Gas demand and supply data at the country level can be found in the supplemental information (S1_data) and for a normal weather year in Tables S7, S8, and S10. COM refers to commercial buildings, IND to the industrial sector, RES to residential buildings, and PWR to the power sector. No changes are recorded for the domestic production of natural gas across weather scenarios. In 2027, total gas demand will decrease by 15 bcm when moving from normal to mild weather, attributable to decreases in heating demand in RES buildings (−7 bcm) and COM buildings (−3 bcm), as well as PWR (−4 bcm). Due to this demand reduction, LNG supply is cut by 11 bcm (Figure 3, bottom panel), while pipeline gas supply is cut by 8 bcm. The remaining difference between supply and demand results from storage accumulations, with storage levels somewhat lower in the mild weather scenario (Figure 3, hydro storage in the top and gas storage in the middle panel).

In contrast, when moving from the normal to the coldest weather scenario, gas demand rises by 23 bcm in 2027 but by 55 bcm in 2031. The increase in demand for natural gas is primarily attributable to higher demand in the power sector, accounting for an additional 9 bcm in 2027 and 36 bcm in 2031, underscoring that the power sector is the primary driver of higher natural gas demand in the coldest winter years. This is due to the displacement of other flexibility sources, like coal-fired PWR, which is pushed out by a rising EU Emissions Trading System (ETS) carbon price. At the country level, when moving from normal to coldest weather in 2031, Germany (+8 bcm), Spain (+8 bcm), the Netherlands (+7 bcm), Italy (+5 bcm), France (+3 bcm), and Belgium (+2 bcm) account for most of the demand increase. Higher heating demand in RES and COM buildings accounts for an additional 18 bcm in 2027 and 2031. The IND sector contributes to some demand savings through DSRs, bridging the remaining difference between the normal and coldest weather year.

To account for the increase in European natural gas demand, in the coldest weather year, the supply of LNG is upped by 20 bcm in 2027 and 39 bcm in 2031. On the contrary, the flow of natural gas through pipelines provides less flexibility. By 2029, the supply of natural gas via pipelines reaches its maximum increase of 13 bcm compared to normal weather, in the coldest year. Most of the increase in pipeline supply is due to higher LNG imports in the UK (+8 bcm), which are then sent via the interconnector to Belgium. Interestingly, pipeline supply falls by 7 bcm in both the coldest and coldest+ scenarios in 2027. This is due to lower gas flows from Ukraine to Hungary, depressed by 3 bcm, and to lower flows from the UK to Belgium and the Netherlands, falling by another 4 bcm, due to higher domestic demand in Ukraine and the UK.

The model includes DSRs for theRESand IND sectors. These are based on price signals computed from demand-side reductions in the EU during the energy crisis of 2021/2023 (see supplemental information SI2 for further details). Table 3 provides an overview of the DSR in the baseline. The mild weather scenario shows no DSR due to higher winter temperatures. Owing to higher natural gas demand and thus higher average prices, the DSR is stronger in the coldest+ year than in the coldest year. Cumulative DSRs in the coldest weather year stand at 14 bcm over 2027–2031, compared to a significantly higher DSR in the coldest*+* weather year of 33 bcm.

**Table 3:** Demand side reductions of natural gas and fuel switching in the power sector

Year	Sector	Normal - Coldest	Normal - Coldest +
2027	RES	1.3	2.8
	IND	5.6	7.0
	PWR	8.5	10.5
2028	RES	0.1	1.8
	IND	3.1	5.8
	PWR	16.2	26.6
2029	RES	0.0	1.0
	IND	1.6	4.3
	PWR	26.4	32.8
2030	RES	0.0	1.0
	IND	0.8	3.9
	PWR	27.9	32.2
2031	RES	0.0	1.4
	IND	1.1	4.0
	PWR	35.8	40.3

Furthermore, with increased renewable energy penetration and lower average gas prices over the modeled period, the DSR becomes less pronounced. For example, in the coldest+ weather year in 2027, 7 bcm is curtailed, compared to only 1 bcm in 2031. The same trend is visible in the coldest scenario, underscoring that the European energy system can better adapt to demand shocks as the share of renewables grows.

Table 3 furthermore depicts fuel-switching in the power sector, computed as the divergence between natural gas consumption in the power sector in the normal weather year and in the coldest/coldest*+*. In fact, the power sector accommodates most of the demand reduction when moving to the coldest and coldest*+* weather years, as coal and hydro power plants become the preferred sources in the merit order. In contrast, in the mild weather year, natural gas consumption in the power sector increases from 4.6 bcm in 2027 to 9.8 bcm in 2030, as lower natural gas prices make it a competitive electricity generation source vis-à-vis coal and hydro.

#### LNG scenarios

After presenting the results of the baseline we now turn to the effects of the sensitivity scenarios. Natural gas demand per LNG scenario under a normal weather year is presented in Table 4. Due to disruptions in the LNG market, cumulative natural gas demand over the period 2027–2031 is lower across all geopolitical scenarios than in the baseline. In the US delay scenario, natural gas demand is 8 bcm lower than in the baseline in 2027 but rises to 2 bcm above it by 2031 due to refilling of gas storage. On average, the demand difference lies at 2.8 bcm per year over 2027–2031. Given the expansion of LNG export capacities in other regions—particularly the Middle East—the impact of delayed US export capacity becomes less pronounced once alternative supply sources become available. In the year of the Strait of Hormuz disruption, both Qatar scenarios show a sharp decline in gas demand relative to the baseline: 18 bcm in QA27 and 9 bcm in QA29. The larger demand reduction in QA27 reflects the earlier timing of the shock, when less new supply is available. By 2029, the additional supply softens the impact. As a result, the disruption to the Strait of Hormuz occurs, the less severe its effect on the European energy system.

**Table 4:** Total natural gas consumption in the LNG scenarios in a normal weather year

Year	Baseline	US delay	QA27	QA29
2027	347	339	329	347
2028	333	331	337	336
2029	321	318	322	312
2030	318	316	315	318
2031	309	311	308	311
Cumulative	1,629	1,615	1,611	1,624

As in the baseline section, the power sector is the primary buffer against supply side shocks in the LNG scenarios.” In the US delay scenario, natural gas use in the power sector declines by 7 bcm in 2027, followed by smaller reductions of 1 bcm in 2028, 2 bcm in 2029 and 2030. The most significant reduction in gas demand for PWR occurs in the QA27 scenario, with a 16 bcm decrease in 2027. However, by 2028, consumption increases by 4 bcm as gas replaces other flexibility sources. This rebound reflects the need to recover hydropower storage levels after a substantial drawdown in 2027. In the QA29 scenario, the reduction in power sector gas demand is more modest, at 8 bcm in 2029, and is preceded and followed by increases in consumption, likely to support flexible management of hydropower storage.

#### Sensitivity scenarios

In the Russian (RU) LNG-only scenario, EU imports of Russian LNG average approximately 26 bcm per year under normal and mild weather conditions. This volume increases to a maximum of 29.5 bcm in the coldest and coldest+ weather years, consistently across 2027–2031. These results suggest that, due to geographic proximity and associated cost advantages, Russian LNG (from Yamal) is a preferred supply source. Compared to the baseline, the increased availability of Russian LNG displaces other suppliers, most notably the US, whose LNG exports to Europe decline by 17–20 bcm over the modeled period. Additional reductions are observed from North Africa (5–8 bcm), Nigeria (1–5 bcm), Trinidad and Tobago (1–3 bcm), and Equatorial Guinea (up to 2 bcm).

In the RU-all scenario, up to 15 bcm of Russian gas is assumed to flow annually to Europe via the Sudzha interconnector through Ukraine. The full volume is utilized across all years and in all-weather scenarios. Compared to the RU LNG-only case, the additional pipeline supply accelerates the displacement of US LNG, with reductions ranging from 17 bcm in 2027 to a peak of 29 bcm in 2030, and 24 bcm by 2031. As in the RU LNG-only scenario, LNG imports from North Africa, Nigeria, Trinidad and Tobago, and Equatorial Guinea also decline. These results highlight the competitive dynamics between Russian and US gas supplies. The added pipeline volumes do not displace Russian LNG, which remains stable at 26–29.5 bcm across weather years. The only exception occurs in 2027 under normal weather, where Russian LNG falls by 3 bcm relative to the RU LNG-only scenario.

Thanks to higher renewable installation rates, gas demand in the REPowerEU scenario drops from 292 bcm in 2027 to 240 bcm in 2031, compared with 347–309 bcm in the baseline. The lower demand is attributable to the power sector, where gas-to-renewable switches replace 51 bcm of natural gas demand over the modeling period in the normal weather year, dropping from 68 to 17 bcm. In return, the baseline sees natural gas demand in the power sector of 120 bcm in 2027 and 83 bcm in 2031.

From the natural gas demand side reaction, we conclude that fuel switching in the power sector offers the biggest lever to reduce European gas demand. Even under the coldest*+* weather scenario in 2031, IND DSR is limited to 4 bcm, primarily through fuel switching. In none of the scenarios modeled here does IND production curtailment occur, in contrast to 13 bcm of production curtailment and 7 bcm of fuel switching in European industries during the energy crisis in 2022.^34^ Lastly, although we see some gas savings in the RES sector, the supply and demand shocks modeled here do not seem sufficient to trigger a stronger response.

### Gas and electricity price reactions

#### Baseline

Table 5 displays average Northwest European gas prices in the baseline scenario. These are computed using the price levels of France, the Netherlands, Belgium, and Germany. For the remainder of this paper, Northwest European gas prices are equated with “EU” gas prices. All modeled prices are updated to Euro 2023 levels. Key assumptions on power demand, installed PWR, and other fuel costs in Europe can be found in the supplemental information (Tables S3–S6). Table S9 displays projected electricity generation and generation capacity in other world regions.

**Table 5:** European average gas and electricity prices in the baseline

	Normal		Mild		Coldest		Coldest+	
	gas	electricity	gas	electricity	gas	electricity	gas	electricity
2027	11.2	145	10.75 (−7%)	138 (−5%)	16.5 (+48%)	179 (+23%)	17.2 (+53%)	182 (+25%)
2028	9.6	136	8.7 (−9%)	126 (−7%)	15.7 (+64%)	177 (+30%)	16.8 (+75%)	182 (+34%)
2029	9.0	132	8.3 (−8%)	124 (−6%)	15.2 (+68%)	173 (+31%)	16.4 (+82%)	181 (+37%)
2030	8.9	128	8.2 (−8%)	122 (−5%)	14.7 (+66%)	169 (+32%)	16.3 (+83%)	180 (+40%)
2031	9.3	130	8.4 (−10%)	122 (−6%)	14.8 (+59%)	168 (+30%)	16.4 (+76%)	179 (+38%)

The price increase in the other weather years relative to the normal weather year is displayed as a percentage. Thanks to an increase in global LNG supply, mainly from the Middle East and the US, as well as to lower natural gas demand due to the EU’s energy transition, the average gas price falls from around 11.2 €/MMbtu in 2027€ to 9.3€/MMbtu by 2031. This figure is significantly lower than the peak price observed during the energy crisis, which reached 59 €/MMBtu in Q3 2022^35^ but remains substantially above the 2019 level of 5.95 €/MMBtu.

In mild winter years, average EU gas prices decline by up to 10% in 2031 relative to the baseline. By contrast, the average gas price increases by 48%–68% under the coldest winter scenario, with an even more pronounced rise of 53%–83% observed under the coldest+ scenario. This asymmetry highlights a key insight: gas prices increase disproportionately under shortage conditions (e.g., coldest*+* scenario) compared to the price reductions observed under loose market conditions (e.g., mild weather scenario), despite the expected substantial expansion in global LNG supply by the end of 2030.

In the coldest and coldest+ weather years, electricity prices rise between 23%–32% and 25%–40%, respectively. Thus, the price increase in the electricity market is not as substantial as in the gas market, suggesting some adaptation potential in electricity production through gas to renewable fuel switching, which will increase over the coming years.

#### LNG scenarios

**Figure 4:**
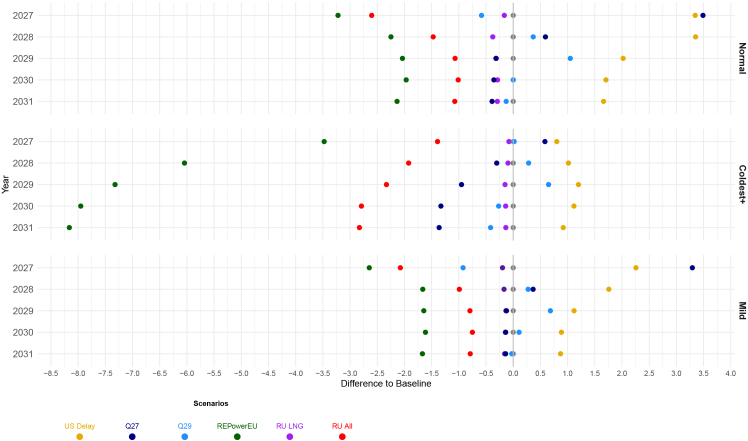
Delta in average gas prices between the baseline and selected scenarios (€/MMBtu)

**Figure 5:**
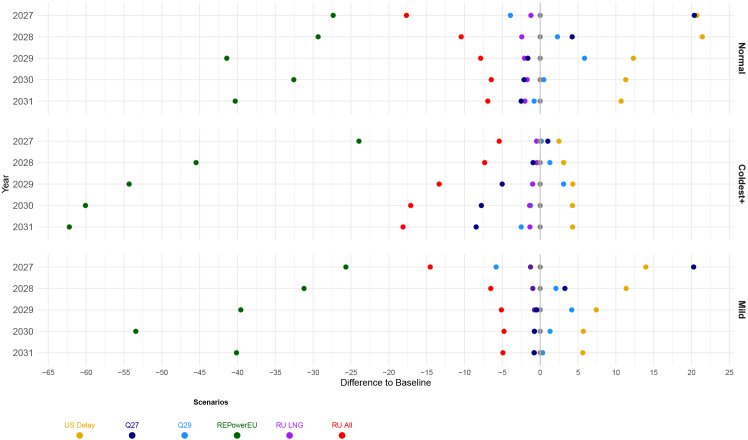
Delta in average electricity prices between the baseline and selected scenarios (€/MWh)

Figures 4 and 5 show the differences in electricity and gas prices between the LNG and sensitivity scenarios, relative to the baseline. In the US delay scenario, gas and electricity prices remain consistently above baseline levels. The average European gas price exceeds the baseline by 1.7–3.4 €/MMBtu (18%–35%), while electricity prices are 11–21 €/MWh (8%–14%) higher. Although these differentials narrow gradually as demand recovers toward 2031, a notable 10 €/MWh gap persists by the end of the period.

The supply shock in the Qatar scenarios is limited to a single year, so its impact on average gas and electricity prices is relatively short-lived. In QA27, the average gas price rises by 3.5 €/MMBtu (31%) in 2027, alongside a 21 €/MWh (14%) increase in electricity prices. However, by 2028, most of this spike is reversed: gas prices fall to 10.2 €/MMBtu, just 6% above the baseline. By 2029, average gas prices in QA27 drop to 4% below the baseline, reflecting demand destruction in non-European regions that frees up additional supply for Europe.

In QA29, average gas prices rise by 1.1 €/MMBtu (12%) in 2029, a more moderate increase than the 3.5 €/MMBtu spike observed in QA27. This results in a smaller increase in electricity prices, at 6 €/MWh (4%). By 2030, both gas and electricity prices return to baseline levels and fall slightly below them by 2031. These findings suggest that the later a potential disruption to the Strait of Hormuz occurs, the more limited its impact on the European energy system, as increased LNG supply from North America becomes available over time.

In this context, one key observation is that, after the shock year in the Qatar scenarios, average gas prices fall within 1–2 years to levels in the baseline. A large-scale disruption of Qatari LNG exports would drive up gas prices worldwide, thus leading to a notable reduction in global gas demand. Between 2027 and 2031, under a normal weather year in Europe, global gas demand is projected to be 14 bcm lower in the QA27 scenario and 47 bcm lower in the QA29 scenario than the baseline. Although the majority of demand destruction occurs within Europe in the shock year—18 bcm in QA27, accounting for 53% of the global demand response (34 bcm), and 9 bcm out of 29 bcm in QA29 (31%)—a non-negligible share also takes place in other world regions. These dynamics suggest that a global supply shock may indirectly support Europe in subsequent years by lowering global demand and easing price pressures, even though Europe bears most of the immediate adjustment burden. Similar observations hold for the European average electricity prices, as shown in Figure 5, given the marginal price-setting of gas-fired power plants.

Unlike the European energy, crisis in 2021–2023, in which MS saw yearly average electricity prices well above 200 €/MWh, in LNG shock scenarios, the average European electricity price does not surpass 200 €/MWh. Especially in the coldest*+* weather scenario, price increases relative to the baseline are relatively muted, given already high power prices in the baseline and the flexibility options of fuel switching from gas to coal. The highest power prices are recorded in the US LNG and coldest*+* scenario at 185 €/MWh throughout 2027–2029.

#### Sensitivity scenarios

A continuation of LNG imports from Russia, worth up to 30 bcm, is unlikely to have a significant positive impact on the average gas price in the EU, leading to a reduction of between 0.2 and 0.4 €/Mmtbu (minus 1%–4%) in a normal weather year. In contrast, the restart of gas supply via the Ukraine transit would ease average gas prices by up to 2.8 €/MMbtu (17%) in 2031, with the most significant impact in the coldest+ winter years, underscoring the need for additional gas import sources or higher DSRs.

If the REPowerEU expansion targets were met (Table 6), this would significantly impact the European gas prices. As shown in Figure 4, average gas prices would be reduced by at least 1.6 €/MMbtu (20%) in 2027, the mild winter year, and by up to 8.2 €/MMbtu (50%) in 2031, the coldest*+* year. Thus, just as with restarting Russian gas imports, the price relief impact is highest in the coldest+ weather scenario, whereas it is less pronounced in mild and normal weather years.

**Table 6:** The EU’s 2030 wind and solar policy targets

	NECP 2019 National Targets	REPowerEU (2022)
Installed wind capacity (GW)	401.0	510.0
Installed solar PV capacity (GW)	378.0	592.0

REPowerEU also proves effective in confining the impacts of LNG shock scenarios on the European energy system. Although not shown in Figure 4, when combining the installed renewable energy capacities of REPowerEU with the three LNG shock scenarios, prices relative to the baseline remain lower under both REPowerEU and LNG shock conditions. For example, in 2028, prices fall by 1.6 €/Mmtbu relative to the baseline for the US delay scenario in a normal weather year once REPowerEU capacities are implemented. Consequently, higher renewable energy capacities would help the EU prepare for the phase-out of Russian energy and benefit the European energy system in case of supply side shocks.

Several conclusions can be derived from the results presented in this section. First, the analysis shows that stopping Russian LNG imports (up to 30 bcm) would have little impact on European wholesale gas prices, provided global LNG supply expands as anticipated. Reintroducing Russian LNG would only lower prices by a marginal €0.2/MMBtu (2%). However, restoring limited Russian pipeline gas flows via Ukraine (up to 15 bcm) could reduce average gas prices by up to €2.8/MMBtu (23%). Still, the price benefits fade by 2031 as global LNG supply expands, aligning with insights from Chyong and Henderson.^36^ Politically, resuming Ukrainian transit would require Ukraine’s consent and may face strong opposition from MS such as Poland and the Baltic states. Although improbable, the EU might reconsider this option post-war if global markets tighten, under strict conditions tied to compliance with peace and capped volumes.

Second, renewables are increasingly driving resilience to gas price shocks. The REPowerEU scenario reduces gas prices by €8.2/MMBtu (21%) and power prices by €62/MWh (30%) in 2031 under the coldest*+* year. These savings are system-wide, not just marginal, indicating that accelerated deployment of renewables is a strategic macro hedge against global gas supply volatility.

Third, we see a high cost of delayed US gas supply. Compared to more transient price shocks in the Qatar scenarios, the US LNG delay scenario leads to a persistent price premium of up to €3.4/MMBtu (35%) over 5 years. This suggests that delays in supply infrastructure outside Europe can impose long-term price burdens, unlike short-lived geopolitical disruptions. It also reaffirms the pivotal role of US exporting projects in shaping the global LNG market over the next few years.

Fourth, we find symmetric price impacts of LNG shocks. While mild weather reduces gas prices by up to 10%, coldest*+* years increase prices by up to 76% (€11.2–17.2/MMBtu). This asymmetry in price response underscores the importance of managing downside risks rather than focusing solely on average trends.

### Behavior of flexibility sources

#### Baseline

Figure 3 presents the utilization patterns of three key sources of flexibility in the EU: hydropower storage, underground gas storage, and LNG import terminals. To compute hydropower storage levels, reservoir and open-loop hydro are used. Due to the fluctuating nature of run of river hydro, this PWR source was discarded. Closed-loop hydro was excluded due to its limited capacity. Seasonal variations are evident, particularly for hydro and gas storage, which provide additional electricity generation or gas supply during periods of elevated demand.

Hydropower storage levels typically peak between May and October, reaching up to 10 bcm-e across Europe. Bcm-e is computed using an average electric efficiency of a combined cycle gas turbine (CCGT) power plant of 55% and respecting a TWh to bcm conversion rate of 11.1. Unlike the previous regional focus on hydropower storage, we also draw on non-EU countries, notably Norway and Switzerland. These two countries have large hydropower storage capacities and can offset demand-side shocks in the EU through electricity exports.

The largest hydro capacities are in Norway, Sweden, Switzerland, Italy, and France. Storage levels are lowest in March, often between 0.3 and 1 bcm-e. Seasonal fluctuations between winter and summer months within a given year reach up to 6 bcm-e. Across all weather scenarios, hydro storage is drawn down more intensively during the early years of the simulation, when global gas markets remain tight. It begins to recover after 2029 as new gas supply projects in the Middle East and North America come online.

Underground gas storage also follows a pronounced seasonal cycle, with levels dropping to around 20 bcm between January and March and rising to as much as 104 bcm by October. Yearly average storage levels are lowest in 2027 across all scenarios: 53 bcm in the mild, 54 bcm in the coldest, 55 bcm in the normal, and 56 bcm in the coldest+ scenario. In the mild scenario, more gas is withdrawn in 2027 due to lower expected demand in 2028, whereas in other scenarios, average levels remain slightly higher. By 2031, annual averages increase to 61 bcm (mild), 60 bcm (normal), and 57 bcm in both the coldest and coldest+ scenarios.

Although hydro and gas storage provide necessary seasonal balancing, LNG inflows through EU terminals are the primary source of flexibility during extreme weather conditions. LNG utilization is substantially higher in the coldest and coldest+ scenarios. Monthly imports range from 12 to 15 bcm in the mild and normal scenarios, compared to 17–20 bcm in the coldest and coldest+ years. For instance, in 2029, the mild scenario sees 150 bcm of LNG imports, while the coldest*+* scenario reaches 226 bcm, a 76 bcm difference in a single year. Furthermore, while the mild and normal scenarios experience a decline in monthly LNG inflows over time (from 15.4 to 13.1 bcm/month), the coldest and coldest+ scenarios attract rising LNG volumes, despite declining total gas demand.

We compute the coefficient of variation for each source in the baseline scenario to assess the contributions of flexibility. The coefficient is defined as the ratio of the monthly standard deviation to the mean across the modeling period (2027–2031). Hydro storage exhibits the highest variability (0.54), followed by underground gas storage (0.38) and LNG terminals (0.34). The high variability of hydro suggests frequent utilization to balance system fluctuations, making it a highly flexible resource in the short term. However, within a cost-minimizing model framework, this implies that hydro is often used at the margin—deployed only after lower cost options are exhausted (i.e., LNG imports and gas storage). This aligns with the logic of a merit-order or flexible supply cost curve, where higher variability may indicate a relatively higher marginal cost of provision. By contrast, while exhibiting lower overall variability, LNG offers substantial upside potential during stress years. Its relatively stable base utilization combined with large import capacity, positions LNG as the EU’s key backstop option under extreme conditions, complementing the seasonal balancing functions of hydro and gas storage.

#### LNG scenarios and sensitivities

**Figure 6:**
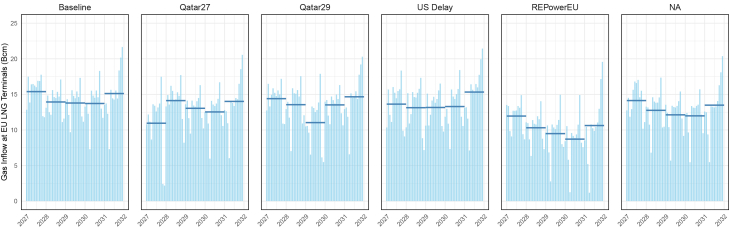
Monthly inflow of LNG at EU terminals in the LNG shock and sensitivity scenarios under a normal weather year

Figure 6 shows the gas inflow at the European LNG terminals across the baseline and the five sensitivity scenarios in a normal weather year. As expected, LNG import volumes drop significantly in 2027 and 2029 in the two Qatar shock scenarios. However, the drop is more severe in Q27, hitting its lowest point at a monthly LNG inflow of 2.2 bcm in December 2027, whereas in Q29 import volumes are lowest in December 2029 at 5.5 bcm. This compares to the average inflow rates of around 10 bcm in December 2024.^37^

With greater expansion of renewable energy sources, annual average LNG inflows fall from 12 bcm in 2027 to 8.7 bcm in 2030, then slightly increase to 10.6 bcm in 2031. The increase in 2031 is owed chiefly to higher import volumes in the Netherlands (+10 bcm annually), while pipeline supplies remain constant. The supply increase is used to fill up gas storage in Germany, Italy, Austria, and France, injecting 7, 4, 1.5, and 3 bcm less into the market, respectively.

From the modeling results, we conclude that hydropower and underground gas storage provide strong seasonal balancing, with hydro peaking at 10 bcm-e in summer and falling as low as 0.65 bcm-e in winter in the Q29 scenario in 2029. Gas storage levels range from 20 to 104 bcm annually. However, LNG terminals emerge as the principal shock absorber under extreme weather, enabling rapid scale-up, such as a 76 bcm increase in 2029 between mild and coldest+ scenarios. Despite hydro showing the highest variability, its relatively high marginal cost means it is used as a last resort. LNG’s large capacity makes it the EU’s most responsive flexibility source in times of stress.

### Country-level energy resilience across EU MS

#### Key characteristics of MS

In Europe, the energy landscape is unevenly distributed, as each MS exercises its right to determine its national energy mix according to Article 194 of the Treaty on the Functioning of the European Union (TFEU). France, Norway, and Sweden account for just under 50% of the region’s total nuclear and hydropower capacity (with France accounting for ∼30%). Hydropower distribution is sparse, with 14 countries having direct access to more than 1 GW of capacity. Germany and Poland are still reliant on coal-fired PWR, generating 104 and 91 TWh of electricity, respectively, in 2024.^38^ The German government seeks to phase out 30 GW of coal capacity over the next few years, requiring investments in up to 20 GW of flexible gas-fired power plants.^39^

**Figure 7:**
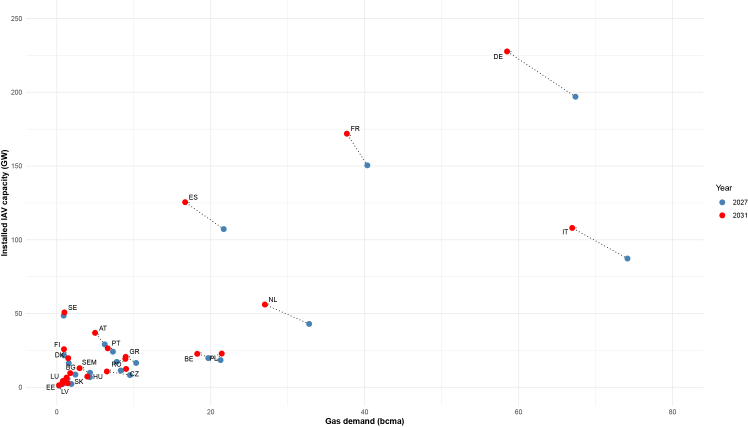
Countries’ relative exposure to gas and electricity shocks using demand data for a normal weather year

Figure 7 represents countries’ inter-annual variation (IAV) capacity and total gas demand (across all sectors) as indicators of risk exposure to gas market shocks. IAV includes PWR from wind, solar, nuclear, and hydro; wind, solar, and hydro are subject to inter-annual variability due to climate conditions, while nuclear, a stable baseload technology, is subject to technological risks, including those induced by weather—droughts—affecting duration of its maintenance and extent of its unavailability. The gas demand data portrayed reflects the underlying assumptions of a normal weather year. Italy records the highest demand for natural gas both in 2027 (74 bcm) and 2031 (67 bcm), of which around half is attributable to natural gas consumption in the power sector, in which 40 GW of gas power plants are installed in 2027, and a further 25% to RES gas demand. Gas demand drops by 8 bcm over 4 years, not least due to an increase in IAV installed capacity from 87 GW in 2027 to 108 GW in 2031, of which hydro makes up a significant proportion at 19 GW. Still, given its large fleet of gas power plants, which are essential to Italy’s security of supply, the country is heavily exposed to supply side shocks (Figure 7).

Germany is the EU’s second-largest natural gas consumer, accounting for 67 bcm of demand in 2027. Like in Italy, natural gas demand in the German market falls by around 9–58 bcm in 2031. Under the normal weather scenario, coal-fired PWR falls from 156 TWh in 2023 to 0 TWh by 2031. However, in a coldest*+* weather scenario, coal PWR in Germany falls only from 274 TWh in 2023 to 65 TWh in 2031, underlining the critical role of coal-fired PWR as a reserve electricity source in Germany.

France is the third-largest consumer of natural gas in Europe, with consumption of 40 bcm in 2027 and 38 bcm in 2031. Between these years, the country’s installed nuclear PWR capacity drops slightly by 2 GW. In contrast, the expansion of onshore (+7 GW), offshore wind (+2 GW), and solar (+15 GW) accounts for most of the increase in IAV capacity, from 150 to 172 GW. Poland is the only major EU MS to see an increase in gas demand, from 19.7 in 2027 to 21.4 bcm in 2031. Most of this demand increase is attributable to an expansion of IND consumption of natural gas.

#### Demand elasticity at the national level

**Figure 8:**
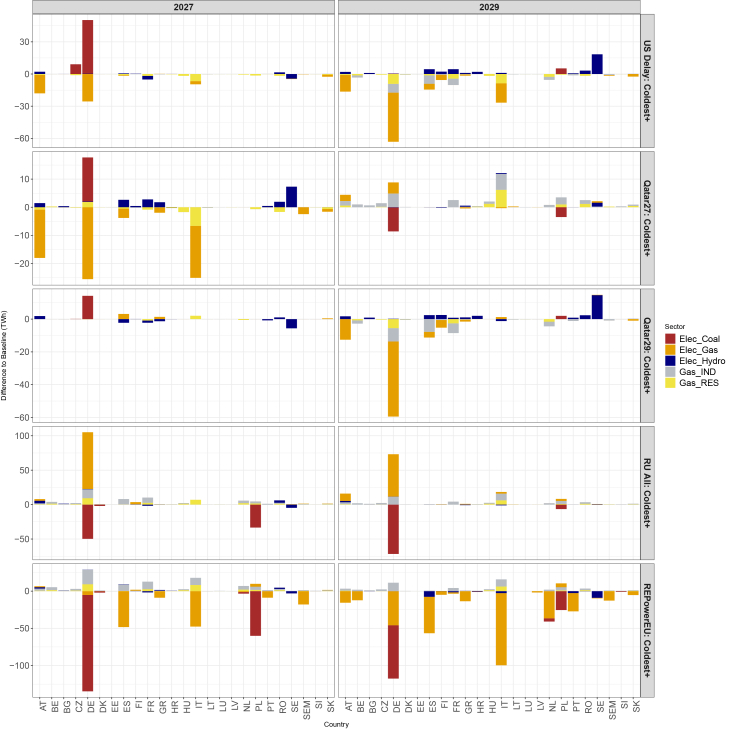
Changes in energy consumption in the electricity and gas markets across European Member States in the coldest+ weather scenario (TWh)

Figure 8 depicts a more detailed breakdown of changes in energy consumption from the baseline scenario to the coldest+ weather across the various LNG and sensitivity scenarios. Differences are shown for 2 years, 2027 and 2029, for the gas market, focusing on IND andRES DSR, and for the electricity market, concerning flexible generation sources such as coal, gas, and hydro storage.

Some countries, including Poland, the Czech Republic, and, most importantly, Germany, have significant potential for fuel-switching between gas- and coal-fired PWR. In scenarios with lower average gas prices (Figure 4), these countries switch to gas-fired PWR, particularly in those with Russian gas imports. In contrast, coal power is preferred in the LNG shock scenarios. Conversely, MS with favorable hydropower potential, i.e., Austria, Spain, Finland, France, Romania, and Sweden, can flexibly draw on their hydro storage reserves.

One key finding is that, although REPowerEU reduces overall gas demand by a cumulative 336 bcm over the model period (2027–2031), it has some opposite effects on gas consumption in industry and RES sectors in the coldest*+* weather year. In 2027, in the IND sector, gas demand rises in Germany (19 TWh), Italy (10 TWh), France (10 TWh), and Spain (8 TWh) if REPowerEU is combined with the coldest+ weather year. Gas consumption in theRESsector in Italy (8 TWh) and Germany (9 TWh) also rises. Fuel switching from coal, particularly in countries such as Poland, the Netherlands, and Germany, and from natural gas to renewable energy sources, more than offsets the increase in demand. As a result, the implementation of REPowerEU would concentrate natural gas consumption in essential sectors, such as industry and residential heating.

#### Variation of gas and electricity prices at the country level

**Figure 9:**
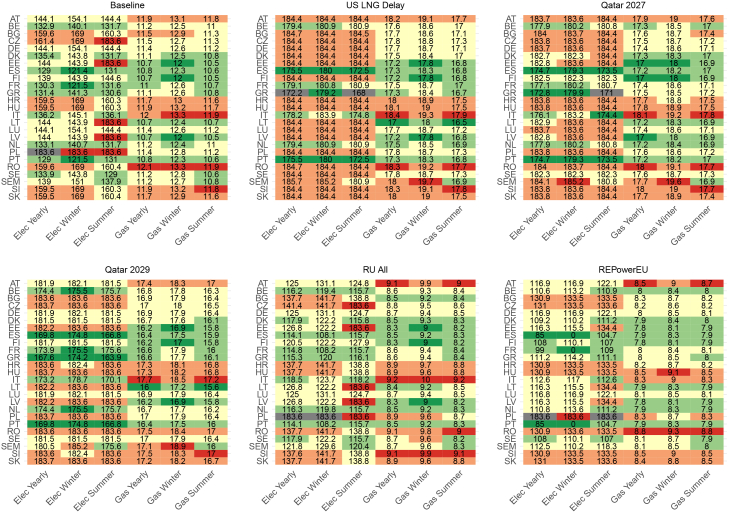
Electricity (€/MWh) and gas prices (€/MMbtu) in 2027 normal weather on the Member State level

**Figure 10:**
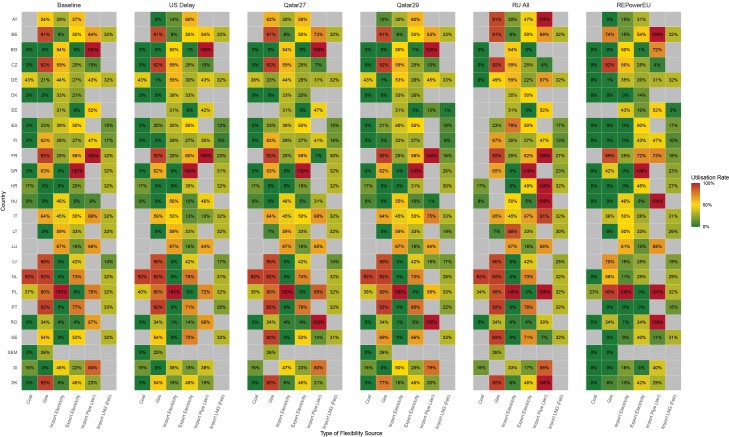
Utilization rates of flexibility sources in the coldest+ weather year for 2029 Yearly average utilization rates were calculated for coal, gas, electricity imports, and electricity exports. For import pipelines, utilization rates are reported for January, which is identified as the month with the highest average pipeline usage. LNG terminal utilization rates are presented for February, which has the highest average LNG import activity.

Figure 9 depicts electricity and gas prices for 2027, on a yearly average and for selected summer (August) and winter (December) months, at the MS level for the normal weather year. Across scenarios, MS in Eastern and Southern Europe, including Bulgaria, Czechia, Hungary, Croatia, Romania, Slovenia, and Slovakia, have the highest electricity prices. This is mainly related to these countries’ limited interconnection capacities, which limit their ability to participate in the EU electricity market (Figure 10). Interestingly, in the US delay scenario, differences in average electricity prices across the EU are relatively muted, ranging from 176 €/MWh in Greece to 189 €/MWh in Ireland, compared with more than 50 €/MWh in the baseline.

Average gas prices are highest in several countries without LNG terminals, including Austria, Hungary, Slovenia, Romania, and Slovakia. On the contrary, Finland and the Baltic countries benefit from low average gas prices thanks to a combined LNG import capacity of 18 bcm, of which 6 are attributable to Finland and 4 to each of the Baltic MS. Italy is a MS that is very vulnerable to gas price shocks, with a premium of 0.4 €/MMbtu in the US delay scenario, compared to the European average. Although this underlines the urgency for the government in Rome to diversify its electricity supply away from gas toward renewables and nuclear, the government has not yet set a target to phase out natural gas in the power sector.^40^ Although average gas prices in Poland tend to fall within the range, electricity prices remain high at 187 €/MWh, even in the REPowerEU scenario. This result is likely due to Poland’s heavy reliance on coal-fired power and high ETS prices (see supplemental information 1, data inputs), which keep power prices constant at around 187 €/MWh.

#### Infrastructure bottlenecks at the member state level

Figure 10 presents average electricity interconnector utilization rates for import and export flows in 2029. The underlying assumption regarding infrastructure build-out can be taken from the Tables S11–S13. Yearly averages were computed for coal, gas, electricity, and import utilization rates. In contrast, January’s utilization rate was selected for using cross-country pipeline infrastructure, while February was identified as the month with the highest average utilization of LNG import infrastructure. The latter choice is due to the natural gas infrastructure experiencing high utilization this month amid cold temperatures. Gray squares appear when a country lacks flexible sources.

Under the supply shock scenarios, Poland exhibits the highest electricity import utilization, reaching 100%. Other MS with substantial import needs include the Czech Republic, Germany, Bulgaria, Hungary, Luxembourg, Slovenia, and Latvia, each with average utilization rates around 50%. On the export side, Greece (100%), Portugal (78%), the Netherlands (73%), Austria (60%), France (60%), and Spain (50%) show high export utilization. In the REPowerEU scenario, overall utilization of cross-border infrastructure declines, reflecting reduced import dependency.

Gas pipeline import flows for January 2029 reveal high utilization rates in several MS. Bulgaria, Romania, and France each operate their pipeline import capacity at 100%. France maximizes its imports from Spain while recording no flows from Belgium. If no flows are recorded from one interconnector, the values are assumed to be missing to reflect infrastructural bottlenecks at other interconnectors. Romania relies exclusively on the pipeline from Hungary, which is also used at full capacity. Other countries with high utilization rates include Italy, Poland, Slovenia, and Belgium. In the Q29 scenario, Italy imports gas from Slovenia at 100% capacity and uses the Croatia interconnector at 50%. These high utilization rates, particularly among Eastern and Southern MS, such as Bulgaria, Greece, Poland, the Czech Republic, Croatia, Hungary, and Slovakia, highlight the need for future investment in transmission infrastructure, storage, and demand-side flexibility. If Russian pipeline imports via Ukraine were to resume, import pipeline utilization would reach 100% in additional countries, including Croatia, Hungary, Slovakia, and Poland, indicating renewed east-to-west gas flows within Europe.

On the other hand, Europe’s LNG import infrastructure seems well-sized to adapt to any future supply side shocks. Across the MS, the utilization rates of LNG import infrastructure in February, the month with the highest average utilization rate, do not exceed 35% in the coldest*+* weather. Croatia and Poland are the countries that see the highest utilization rate of their LNG import terminals in 2029 and the coldest*+* weather, at around 34% in the Q29 scenario.

Some LNG infrastructure is underused, such as in Latvia, according to the baseline (13%). It is interesting to see that, across scenarios, LNG terminals show roughly the same (low) utilization rates in 2029, underlining the relatively constant LNG inflow. Only by 2031, Finland and Sweden’s LNG terminals show utilization rates above 50% in December across some weather years. This month, they can be as high as 90% in Finland under the scenarios REPowerEU and coldest+, REPowerEU and coldest, REPowerEU and normal, and baseline and mild. In these scenarios, more natural gas is supplied to Eastern Europe via LNG terminals in Finland and Sweden, replacing potentially more expensive pipeline imports.

In this analysis, we assumed that Germany has three LNG terminals (Brunsbüttel, Stade, and Wilhelmshaven), each with an import capacity of 7.5 bcm. Thus, Germany has a total import capacity of 22.5 bcm per year, which will be fully operational by 2027. In 2031, the utilization rate of German LNG terminals reaches its recorded maximum in the coldest+ weather year, at 38%. In reality, thanks to floating storage and regasification units (FSRUs), the injection capacity is set to reach 28 bcm by 2025, expand to above 40 bcm by 2030, and fall to 36 bcm by 2035.^30^ Given the low utilization of German LNG terminals indicated by this modeling analysis, the country will likely overbuild its LNG import capacity, making it a stranded asset for its taxpayers.

In light of these results, we can conclude that there is a risk of under-utilization of high-cost LNG infrastructure. Terminals across Europe rarely exceed 35% utilization, reaching up to 90% only in specific months and countries, such as in Finland. Notably, German LNG terminals reach only 38% utilization in the coldest*+* year by 2031. This calls into question the ongoing expansion to over 40 bcm, especially when modeling suggests the infrastructure will not be needed at such a scale even under extreme stress scenarios. Separately, we find that MS in Eastern and Southern Europe exhibit lower energy resilience due to the lack of cross-border transmission capacity for both gas and electricity. Additionally, the existing European LNG terminal and FSRU capacity projected for 2031 (286 bcm) is largely concentrated in richer Western and Northern MS, with the Eastern and Southern regions accounting for only 16% of European terminal and FSRU capacity. Thus, these regions are more dependent on the continuity of pipeline flows or cross-border imports from their European neighbors.

## Discussion

The modeling suggests that by 2031, Europe can generally maintain physical supply under compound gas-electricity stress through a portfolio of flexibility options: underground gas storage, hydropower storage, LNG imports, interconnectors, fuel switching in PWR, and some DSR. However, energy resilience should be expressed both in terms of “keeping the lights on” and, importantly, in terms of limiting tail-price outcomes. Price responses are highly non-linear across weather years: gas prices rise by 48%–83% in coldest/coldest+ conditions while falling by only 7%–10% in mild years, with electricity prices rising by 23%–40%. This asymmetry indicates that system performance under multi-week scarcity cannot be inferred from static adequacy checks, and supports the case for a composite resilience metric that stress-tests jointly across vectors, time horizons, and networks.

The results also indicate where policy attention is likely to be most productive. LNG is the primary shock absorber in extreme conditions, e.g., a 76 bcm swing in 2029 between mild and coldest+; yet many terminals remain underutilized, while electricity and gas interconnectors in Eastern and Southern MS frequently reach high utilization rates. This combination points to a need to target infrastructure investment toward binding network constraints and cross-border deliverability, while scrutinizing additional LNG build-out where utilization remains modest even in stress years.

On the demand side, the model includes IND andRESDSR but shows it remains limited relative to the scale of weather-driven demand increases, and no IND production curtailment occurs across scenarios. Given that price spikes are nevertheless severe (e.g., around 182 €/MWh in the coldest+ year in 2027), institutionalizing DSR as a recognized flexibility resource would strengthen the adjustment margin, thereby reducing reliance on expensive scarcity hours.

Finally, accelerated deployment of renewables materially reduces exposure to gas-linked scarcity. In an accelerated renewables deployment pathway (such as REPowerEU), lower gas use in the power sector delivers sizable price relief, particularly in the most stressed weather years, consistent with renewables acting as a macro hedge against LNG-market volatility. Since gas-fired generation continues to set marginal prices under tight conditions, the case for complementary long-term contracting follows directly: expanding contracts for difference (CfDs)/Power Purchase Agreements (PPAs) in power and coordinated procurement/longer-term contracting mechanisms in gas reduce the pass-through of short-term gas volatility to electricity prices and end-users, without relying solely on additional physical capacity.

At the beginning of this research paper, we posed the following question: “how resilient is the European energy system to compound supply and demand shocks and what role can market design and policy tools play in mitigating these risks?” We find that by 2031, Europe’s energy system is increasingly well-equipped to absorb HILP events through existing flexibility mechanisms, including underground gas storage, hydropower storage, LNG terminals, electricity and gas interconnectors, and DSRs. In none of the scenarios modeled in this study does IND production curtailment occur, in contrast to 13 bcm of production curtailment and 7 bcm of fuel switching in European industries during the energy crisis in 2022.^34^ Nevertheless, electricity price spikes remain severe, averaging 182 €/MWh in the coldest+ year (2027), double current wholesale levels, likely forcing some IND facilities to reduce output and derail the EU’s push for increased economic competitiveness. Informed by our modeling results, we outline five priority policy areas to improve resilience against HILP events.

### Establish a composite resilience metric to integrate stress testing across the European energy system

Our modeling demonstrates that existing adequacy metrics, focused on *N*-1 security or peak-load sufficiency, are insufficient to evaluate the resilience of the European energy system under compound or cross-vector stress events. We therefore recommend developing a composite resilience metric (CRM) that jointly assesses seasonal adequacy, infrastructure-failure tolerance, and inter-annual variability in renewable output and demand.

Our modeling results show that conventional adequacy indicators fail to capture key drivers of resilience under compound shocks. Price reactions are strongly asymmetric across weather years: in the coldest and coldest+ cases, average EU gas prices rise by 48%–83% and electricity by 23%–40%, whereas in mild years prices fall by only 7%–10% (Table 5). This non-linear behavior reflects sustained multi-week scarcity, where prolonged cold periods and limited inflows deplete storage and flexibility reserves faster than they can recover. Such cumulative stress dynamics cannot be captured by single-hour adequacy or *N*-1 contingency assessments, which measure only instantaneous capacity margins. Cross-vector adjustments are equally important: the power sector becomes the primary buffer, with large gas-to-coal and hydro switching and materially different DSR volumes across years (Table 3), while pipeline flexibility saturates (e.g., maximum additional pipeline supply ∼13 bcm in 2029 under coldest conditions). Infrastructure stress is also spatially uneven: LNG acts as a backstop in stress years (226 bcm vs. 150 bcm in 2029 coldest+ vs. mild), yet many LNG terminals remain under-utilized, while electricity and gas interconnectors in Eastern and Southern Europe frequently reach 100% utilization (see “behavior of flexibility sources” to “country-level energy resilience aross EU MS”; Figure 10). These dynamics show that resilience must be assessed through multi-week energy sufficiency, network congestion under joint stresses, and realized flexibility responses rather than *N*-1 or peak-snapshot indicators.

The forthcoming revision of the EU energy-security framework,^41^ including updates to the Gas Security of Supply Regulation (EU) 2017/1938 and the Risk-Preparedness Regulation (EU) 2019/941, provides an opportunity to revise and codify such a metric. Thus, these acts form the legislative backbone of the Union’s crisis-management regime and could be amended to include resilience-performance indicators within mandatory risk-assessment methodologies. The European Network of Transmission System Operators for Electricity (ENTSO-E) and the European Network of Transmission System Operators for Gas (ENTSO-G) could operationalize the metric within their existing adequacy and risk-assessment frameworks. To ensure that resilience considerations inform investment and planning decisions, the CRM should also be embedded in EU-level cost-benefit and network-development assessments, notably the Ten-Year Network Development Plan (TYNDP) and the Projects of Common or Mutual Interest (PCI/PMI) process governed by the Trans-European Energy Networks (TEN-E) Regulation (EU) 2022/869. Integrating CRM-based indicators into these processes would enhance transparency, prioritize flexibility assets with the most significant systemic value, and enable more coordinated, forward-looking responses to HILP shocks.

### Target infrastructure investment where it adds system resilience

The modeling reveals marked differences in infrastructure utilization across regions: while some countries face near-full utilization of pipelines and interconnectors, others risk stranded LNG assets. Public support for new energy infrastructure should, therefore, prioritize projects that demonstrably enhance systemic resilience rather than merely increase nominal capacity. In particular, investments should focus on electricity and gas interconnectors in Eastern and Southern Europe, where physical and regulatory constraints limit responsiveness to shocks, while reassessing LNG terminal expansion in regions such as Germany and Italy,^42^ where utilization remains modest even under severe conditions.

The Action Plan for Grids^43^ and the forthcoming European Grids Package^44^ provide a coherent legislative framework for prioritizing infrastructure projects that deliver measurable resilience benefits by accelerating cross-border interconnections, reducing permitting delays, and enabling anticipatory investments. Within the TEN-E Regulation (EU) 2022/869,^45^ the Projects of Common and Mutual Interest (PCI/PMI) process should apply resilience-based criteria consistent with the CRM, ensuring that the infrastructure pipeline reflects both adequacy and adaptability objectives.

Access to public financial support should also be conditional on demonstrated system-level benefits. The Clean Industrial Solutions Aid Framework (CISAF),^46^ adopted under the Clean Industrial Deal, provides a mechanism for supporting strategic investments in grids, storage, and flexibility assets where market revenues are insufficient to recover costs. Applying CISAF criteria to cross-border and flexibility-related projects would help direct public funding toward assets that genuinely strengthen system resilience, avoiding regional overcapacity or stranded investments.

### Institutionalize DSR as a recognized flexibility resource

Demand-side flexibility, particularly from industry, plays a measurable role in moderating gas and electricity demand shocks, yet its full potential remains underutilized. To unlock this potential, the European Union’s Electricity Market Design reform, adopted in 2024 through Directive (EU) 2024/1711 and Regulation (EU) 2024/1747, should be implemented by MS in a way that institutionalizes DSR as a core flexibility resource within market operation and adequacy planning.

The reform explicitly enables broader consumer participation by establishing rules for aggregator access, dynamic-pricing contracts, and PPAs that reward flexibility. MS should (1) adopt aggregation frameworks with transparent participation thresholds and remuneration mechanisms, (2) expand the deployment of smart metering and dynamic-tariff structures to encourage flexible consumption, and (3) integrate DSR into resource adequacy assessments and emergency planning under the Risk-Preparedness Regulation (EU) 2019/941. Embedding DSR into ENTSO-E and the Regional Coordination Center (RCC) adequacy and crisis-management methodologies would ensure that demand-side flexibility is treated symmetrically with supply side resources, reducing reliance on costly standby generation, enhancing operational efficiency, and strengthening overall system resilience.

### Accelerate renewable energy deployment with priority grid access

Expanding renewable energy capacity, particularly in line with the REPowerEU objectives, significantly mitigates gas and electricity price spikes during extreme conditions. While the ongoing implementation of the Renewable Energy Directive (RED III, Directive [EU] 2023/2413) and the Electricity Market Design reform have created a stronger enabling framework, further acceleration is required to ensure timely delivery and integration. Full and effective transposition of RED III, especially its provisions on go-to areas, permitting time limits, and priority grid connection, is crucial to prevent delays and curtailment.

Building on the Action Plan for Grids (COM [2023] 757) and the Guidelines on Anticipatory Investments,^47^ grid reinforcements and forward-looking network development in high-resource regions should be prioritized to accommodate new capacity and enable renewables to participate fully in balancing and ancillary services markets. Faster renewable integration directly lowers reliance on gas-fired generation during supply stress periods, dampening price volatility and improving both economic and environmental resilience during HILP events.

### Encourage long-term contracting to reduce exposure to gas-linked price volatility

Despite the growing share of renewables, gas-fired power plants continue to set marginal prices under tight market conditions. To mitigate exposure to gas-price volatility, long-term contracting mechanisms must be strengthened across both the electricity and gas sectors. Building on the Electricity Market Design reform, Member States should expand long-term power purchase agreements (PPAs) and two-way CfDs to stabilize investment returns for low-carbon generators and reduce price pass-through to consumers.

On the gas side, the Affordable Energy Action Plan (COM [2025] 79)^48^ institutionalizes the Aggregate EU mechanism as a permanent platform for coordinated gas procurement and long-term contracting. This mechanism enhances Europe’s negotiating position and improves price predictability by aggregating demand and entering into longer term supply agreements. Transparency and integrity in these transactions are further supported by the Regulation on Wholesale Energy Market Integrity and Transparency (REMIT) recast (Regulation [EU] 2024/1106) and the Gas and Hydrogen Markets Regulation (EU) 2024/1789, which together establish a unified framework for renewable and low-carbon gas markets. Aligning electricity, gas, and hydrogen contracting frameworks under these legislative instruments would reduce exposure to short-term volatility in fossil-based gas prices.

### Limitations of the study

The research was conducted using detailed and sophisticated modeling tools. However, some limitations deserve further research. First, the model assumes perfect foresight, meaning that all future system parameters, such as demand, technology costs, and fuel prices, are known to the optimization model. This assumption enables a consistent assessment of long-term equilibria but inevitably simplifies the uncertainty inherent in real-world decision-making. Accordingly, the results should be interpreted as sensitivity analyses and robustness checks rather than deterministic forecasts, illustrating how the system would respond under alternative policy, cost, or resource conditions. Second, because the model is partial-equilibrium, interactions between the energy sector and broader macroeconomic dynamics are not explicitly captured, which may either amplify or dampen gas consumption responses. Third, although the framework represents fuel switching in PWR outside Europe, it does not fully capture the responses of other major gas importers, such as Japan, India, or China, to global supply shocks. These aspects warrant further investigation in future work.

## Resource availability

### Lead contact

Further information and requests for resources and reagents should be directed to and will be fulfilled by the lead contact, Chi Kong Chyong (kong.chyong@oxfordenergy.org).

### Materials availability

This study did not generate new unique materials.

### Data and code availability

• Data reported in this paper will be shared by the lead contact upon request. Processed model data have been deposited within the supplemental information as S1_data (reported in the key resources table) and are publicly available as of the date of publication.
• All original code used in this paper is available from the lead contact upon request.
• Any additional information required to reanalyze the data reported in this paper is available from the lead contact upon request.

## Acknowledgments

The authors gratefully acknowledge David Ah-Voun for his support in visualizing an earlier version of this research as a working paper. His contribution was very valuable in shaping the initial presentation of the study. Further, the authors extend their gratitude to the Center on Global Energy Policy at Columbia University and to the Oxford Institute for Energy Studies for providing funding for this research paper.

## Author contributions

Supervision, conceptualization (lead), methodology (lead), data curation, validation, writing – original draft and writing – review and editing (equal), C.K.C. Formal analysis, methodology (supporting), conceptualization (supporting), visualization, and writing – review and editing (equal), H.S.

## Declaration of interests

The authors declare no competing interests.

## STAR★Methods

### Key resources table

**Table:** 

REAGENT or RESOURCE	SOURCE	IDENTIFIER
**Deposited data**		

Processed model data	This paper	S1_data
Electricity demand	Copernicus Climate Data Store	https://eepublicdownloads.azureedge.net/clean-documents/sdc-documents/ERAA/Demand%20Dataset.7z
Electricity demand	Ah-Voun et al.^49^	https://www.sciencedirect.com/science/article/abs/pii/S030142152300441X?via%3Dihub
Electricity generation mix	ENTSO-E	https://view.officeapps.live.com/op/view.aspx?src=https%3A%2F%2Feepublicdownloads.azureedge.net%2Fclean-documents%2Fsdc-documents%2FERAA%2FPEMMDB%2520National%2520Estimates.xlsx&wdOrigin=BROWSELINK
Electricity generation mix	ENTSO-E	https://www.entsoe.eu/eraa/2021/
Electricity generation mix	ENTSO-E	https://transparency.entsoe.eu/
Network capacity	JRC	https://zenodo.org/records/3574566#.YwKJIHbMIQ8
Network capacity	ENTSO-E	https://transparency.entsoe.eu/
Commodity prices	ENTSO-E	https://transparency.entsoe.eu/
Natural gas demand	Ah-Voun et al.^49^	https://www.sciencedirect.com/science/article/abs/pii/S030142152300441X?via%3Dihub
Natural gas supply	BP	https://www.bp.com/content/dam/bp/business-sites/en/global/corporate/pdfs/energy-economics/statistical-review/bp-stats-review-2022-full-report.pdf
Natural gas supply	NESO	https://www.neso.energy/publications/future-energy-scenarios-fes/fes-documents
Natural gas supply	Chyong and Hobbs^13^	https://www.sciencedirect.com/science/article/abs/pii/S0140988314000905?via%3Dihub
Natural gas supply	Chyong et al.^50^	N/A
Natural gas storage	IEA	https://www.oecd.org/content/dam/oecd/en/publications/reports/2019/09/natural-gas-information-2019_8c332194/4d2f3232-en.pdf
Natural gas storage	EIA	https://www.eia.gov/naturalgas/storagecapacity/
Natural gas transport	IEA	https://www.iea.org/reports/gas-market-report-q3-2022
Natural gas transport	ENTSO-G	https://www.gie.eu/press/system-development-map-2021-2022-published-by-entsog-gie/

**Software and algorithms**		

Partial equilibrium model	This paper	Available from the lead contact upon request.
AIMMS 26.5	This paper	https://www.aimms.com/

### Method details

#### Modeling framework and contribution

The paper contributes to the growing literature that seeks to embed resilience considerations into energy system modeling. Many existing studies have analyzed the consequences of removing Russian energy imports to Europe using macroeconomic or integrated assessment frameworks. Perdana et al.^51^ employ a general equilibrium model to estimate EU-wide welfare losses associated with eliminating Russian gas. They find that a full energy embargo would double European welfare losses relative to an oil-and-coal embargo, while Russia’s welfare loss would rise by 44%. Liu et al.^52^ estimate that a complete gas embargo would reduce EU GDP by 2% and Russian GDP by 5%, and note that a 10% demand reduction - modeled as a demand-side response - is essential to mitigating economic impacts while cutting EU carbon emissions by 10%. Sampedro et al.^53^ use an integrated assessment model to show uneven regional impacts, with gas price increases ranging from +4% in Southwestern Europe to +18% in Eastern Europe. While these studies offer valuable macro-level insights into welfare, GDP, and emissions, they are less suited to capturing the short-to medium-term operational vulnerabilities that determine resilience. Their focus on regional averages also obscures country-level infrastructure constraints and behavioral responses within key energy-consuming sectors.

Partial-equilibrium models, by contrast, provide greater granularity in representing market dynamics, technology substitution, and the flexibility mechanisms that respond to shocks. For instance, Barner et al.^54^ show that EU gas demand could be met without Russian imports by expanding LNG imports from other world regions to cover up to 30% of supply, though at a 10% price premium. The EWI (2025) study simulates risk scenarios for the German gas market, assuming no access to Russian pipeline gas or LNG after 2025. The researchers find that the system can adapt through diversification, with EU gas prices declining from €40/MWh in €2025 to €30/MWh by 2030 as imports from Norway and North Africa increase. McWilliams et al.^55^ similarly show that a full ban on Russian LNG imports would be manageable. However, Hauser^56^^,^^57^ cautions that diversification raises system costs and heightens the risk of regional congestion during extreme weather events. These studies demonstrate the growing use of partial-equilibrium models to explore resilience. However, most remain focused solely on gas, neglecting coupled interactions with electricity systems and the compounding nature of HILP events.

Building on this literature, the present study extends an established partial-equilibrium global energy market model to represent the gas and electricity sectors jointly and to quantify how the European energy system responds to compound HILP shocks in the medium term (to 2030/31) without access to Russian energy. This period coincides with rapid decarbonization and infrastructure transitions as the EU pursues its strategic objective of eliminating Russian gas imports by 2027. What was once a hypothetical scenario has now materialised: Europe has already lost most of its Russian gas supply. Therefore, our analysis assesses how the system can continue to function under this new reality, including extreme weather and global LNG disruptions.

The model is formulated as a quadratic programming problem in AIMMS and solved using the IBM CPLEX solver (see below for the mathematical formulation). It integrates the interactions between global gas and electricity markets, including LNG shipping, pipeline flows, and electricity generation, and explicitly represents supply- and demand-side shocks. Compared with general-equilibrium or IAM frameworks, this structure allows for higher spatial and temporal resolution, capturing country-level infrastructure constraints, technology substitution, and behavioral demand responses. The model builds on established gas-market frameworks (Zwart and Mulder^58^; Holz et al.^59^; Lise and Hobbs^60^; Gabriel et al.^61^; Abada et al.^62^; Chyong and Hobbs^13^; Growitsch et al.^63^) while extending them in several key respects. Previous coupled gas-electricity studies (Abrell and Weigt^64^; Deane et al.^18^) focus mainly on Europe; here, the coverage is global, capturing interactions among gas, coal, oil, and electricity markets. The model also builds on the earlier work of Chyong and Hobbs,^13^ Chyong et al.,^50^ and Chyong and Henderson^36^ by introducing demand-side response and inter-fuel competition dynamics at both European and regional levels.

The extended framework incorporates a detailed representation of the power sector to capture substitution among gas, thermal coal, and renewables. It includes short-term global LNG shipping dynamics across 25,782 routes and seven maritime chokepoints - the Panama Canal, Suez Canal, Cape of Good Hope, Cape Horn, Gibraltar, Malacca, and Northern Sea Route - making it well suited to analyze the resilience implications of maritime disruptions (Libby and Christiansen^65^; Meza et al.^66^). Forty years of hourly European climate data are used to represent inter-annual and spatial variability, including temperature, wind speed, solar radiation, and hydro inflows. This allows the model to simulate weather-driven variations in gas demand for heating and renewable generation. No existing security-of-supply study has incorporated this level of meteorological granularity.

On the demand side, explicit demand-side response mechanisms are introduced for industrial andRESgas consumers. In the industry, the model includes fuel switching and output curtailment triggered at predefined cost thresholds, calibrated in line with IEA^67^ and recent empirical studies (Ruhnau et al.^68^; Moll et al.^69^; Chiacchio et al.^70^). ForRESbuildings, gas demand reductions are estimated using a heating-degree-day model (Ah-Voun et al.^49^) that captures thermostat adjustments in response to high prices. The full implementation is described in Supplementary Information 2.

These innovations allow this study to provide a comprehensive, high-resolution analysis of Europe’s ability to withstand, absorb, restore, and adapt to HILP conditions across gas and power systems. Whereas previous research has typically examined the effects of weather events or infrastructure risks in isolation, this study combines them within a unified resilience framework. It provides a systematic stress test of the European energy system under adverse yet plausible conditions in the critical years leading up to 2030/31. It offers insights into the mechanisms underpinning energy resilience in an increasingly decarbonised and interconnected system.

#### Scenario design and analytical framework

Building on the conceptual foundations presented in Chapter 1 and above, this Section describes how the resilience framework is operationalized through a set of stylized scenarios. The aim is to assess the resilience of the European energy system under a range of plausible yet adverse conditions by observing how economic and technical-physical responses evolve across the stages of the resilience trapezoid: withstanding, absorbing, restoring, and adapting. The analysis draws on lessons from the 2021/23 energy crisis. It uses structured HILP scenarios to test the system’s ability to cope with three main sources of stress: (1) inter-annual weather variability affecting energy supply and demand; (2) geopolitical disruptions to global LNG supplies; and (3) uncertainties surrounding the implementation of European energy and climate policies.

The scenarios are not forecasts but stress tests designed to explore how different physical and institutional shocks may interact to challenge Europe’s energy system. They allow us to identify which flexibility mechanisms, such as storage, fuel switching, demand-side response, or LNG diversification, enhance resilience and maintain stability under stress conditions. In line with the performance-based framework introduced earlier, each scenario produces measurable indicators that reflect economic responses (changes in wholesale prices and demand-side adjustments) and technical-physical responses (utilisation of flexibility sources, storage behavior, and infrastructure flows).

The scenario design begins with a Baseline reflecting current EU policy trajectories and energy-system evolution. Building on this, three scenario clusters are developed: (i) weather-related shocks, (ii) geopolitical LNG supply disruptions, and (iii) policy sensitivities relating to Russian gas imports and renewable-energy deployment. Each of these is evaluated under four stylised weather years - Mild, Normal, Coldest, and Coldest + Drought - to capture inter-annual variation in energy supply and demand. The cross-combination of all cases yields 28 distinct model runs used to stress-test the system over 2027–2031. Table 2 summarizes all scenarios and their weather overlays, with further detail on each scenario cluster outlined below.

#### Research framework and baseline definition

The analytical framework begins by establishing a Baseline scenario that serves as the primary reference point for evaluating all other scenarios. The Baseline reflects the current European policy trajectory and incorporates three key assumptions.

1. Cessation of Russian energy imports (both pipeline and LNG) from 2027 onward, in line with the European Commission’s 2025 legislative proposal;
2. Technology deployment consistent with the 2019 National Energy and Climate Plans (NECP19), representing Member States’ existing commitments for renewable energy capacity by 2030;
3. No disruptions to global LNG supply chains, implying that Europe continues to access diversified LNG volumes on competitive terms.

This Baseline captures a European energy system transitioning away from Russian gas, operating under average hydro and nuclear conditions calibrated to historical data (2016–2021 for nuclear, 2000–2021 for hydro). Each Baseline variant is stress-tested against the four weather years introduced below, allowing identification of flexibility requirements and system vulnerabilities. The other scenario clusters then add exogenous geopolitical or policy-related shocks under the same weather conditions to ensure comparability across results. Complete numerical outputs for all combinations, including those not discussed in detail here, are provided in the accompanying Excel databook.

#### Weather scenarios

Weather is a critical determinant of both energy supply and demand. While wind and solar output variability is often examined at intraday or seasonal scales, their production is also subject to inter-annual fluctuations (see Ah-Voun et al.^49^ and citations therein). Although hydroelectric generation is dispatchable within a given year, it too is exposed to inter-annual variability - drought conditions, for example, can significantly reduce water availability and thus electricity generation potential (see Brás et al.^71^; IEA^67^), as was evident during the 2022 drought in Europe (Jones et al.^72^). These inter-annual variations are becoming increasingly relevant as Europe continues to rely more heavily on renewable energy sources, especially variable renewable energy (VRE), to displace fossil fuels.

Four stylised weather scenarios are constructed to capture the implications of such weather-driven variability based on the clustering of forty years of hourly temperature data across European countries, as developed in Ah-Voun et al.^49^ These scenarios are subsequently used in a Heating Degree Demand (HDD) model to estimate gas demand for space heating in theRESand COM sectors. The model explicitly incorporates price-sensitive demand-side responses in industry andRESbuildings, based on behavioral changes observed during Europe’s energy crisis 2021-23. Wind, solar, and hydroelectric generation resource availability is derived from the Pan-European Climate Dataset (PECD) for each weather year. For a detailed description of the HDD model and the clustering approach used to define the weather scenarios, see Ah-Voun et al.^49^ The four weather scenarios are as follows.

1. *Mild* Winter
2. *Normal* Winter
3. *Coldest* Winter
4. *Coldest* Winter *+* Summer Drought (*coldest+*)

The first three scenarios focus on winter heating conditions. The fourth scenario, *coldest+*, additionally assumes a severe summer drought similar to that experienced in 2022, which significantly affected hydroelectric and nuclear power generation. That year, Europe faced its most extreme drought in at least 500 years,^72^ resulting in a 12% decline in hydroelectric output relative to the 2000–2021 average. Furthermore, technical constraints and water shortages in rivers curtailed the operation of the French nuclear fleet, whose electricity output fell by 30% compared to the same historical average.^73^

Given that climate change is expected to increase the frequency and severity of droughts across Europe,^74^ incorporating such a drought scenario is essential for stress-testing the resilience of the European energy system. For the first three weather scenarios, nuclear generation is assumed to reflect the average output from 2016 to 2021. In the *coldest+* scenario, nuclear output is held constant at 2022 levels, while hydro generation is based on the PECD’s lowest historical inflow year, 1991. This allows us to combine the most severe winter conditions with the most severe summer supply constraints, thereby constructing a compound HILP scenario to evaluate the limits of system resilience.

#### LNG supply-side shock scenarios

Since Russia’s full-scale invasion of Ukraine, the European Commission and the majority of national governments across Europe have committed to phasing out Russian natural gas. In 2024, Russia continued to supply gas to Europe through three principal routes: pipeline flows via Ukraine (17 bcm) and Turkey (16 bcm), and LNG deliveries from the Yamal LNG plant to Northwest and Southern Europe (21 bcm).^6^ These volumes represented 18% of the EU’s gas imports in 2024,^6^ down significantly from 39% in 2021. With the expiration of the Ukraine Transit Agreement at the end of 2024, only two possible routes for Russia to deliver its gas to the European market remain: via TurkStream or via LNG shipments. For a detailed account of infrastructure availability, export routes, and contractual arrangements between Russia and European importers, see Chyong and Henderson.^36^

In line with the REPowerEU Roadmap, the EC issued a legislative proposal in June 2025 to phase out all Russian energy imports by the end of 2027.^8^ Under this framework, European companies are prohibited from entering into new contracts with Russian suppliers, while judicial protections are granted to allow the termination of existing long-term contracts by 2027. The Baseline reflects this policy trajectory by assuming a complete embargo on all Russian gas flows to Europe (excluding Türkiye) - pipeline and LNG - from 2027 onwards.

Beyond this policy-driven embargo, the analysis explores the implications of exogenous supply-side shocks that could further constrain Europe’s access to global LNG markets. The potential for maritime transport disruptions to affect LNG supply has been studied in earlier work, including assessments of the Panama and Suez canals (Libby et al.^65^; Meza et al.^66^), and recent modeling of LNG disruption impacts on the German market.^30^ Building on this literature and current geopolitical developments, three LNG shock scenarios were modeled.

1. Qatar LNG Disruption in 2027 (127 bcma), QA27
2. Qatar LNG Disruption in 2029 (127 bcma), QA29
3. US LNG Expansion Delay (40-45 bcma, 2027–2031), US Delay

#### Qatar LNG disruptions (2027 and 2029)

In the wake of escalating tensions in the Persian Gulf, triggered by aerial bombardments of Iranian facilities by American forces, the Iranian parliament called for the closure of the Strait of Hormuz on June 22, 2025. While the military feasibility of such a closure remains uncertain, the analysis assumes a complete one-year disruption of LNG traffic through the Strait. Given that approximately 20% of global LNG exports pass through this strategic chokepoint, such a disruption would remove around 127 bcma of Qatari LNG from global markets, equivalent to the EU’s entire LNG demand in 2024.^37^ Although 86% of Qatari LNG in 2024 was shipped to Asia and only 14% to Europe,^75^ the shock would reverberate globally through elevated prices and forced demand destruction. To explore the sensitivity of outcomes to the timing of such a disruption, this scenario is implemented for 2027 and 2029.

#### US LNG expansion delay (2027–2031)

A third scenario considers the risk that planned expansions in US LNG export capacity fail to materialize during the second half of this decade. Although a complete cessation of US LNG exports to Europe is considered highly unlikely - even in intensifying transatlantic trade tensions - the prospect of delays or cancellations of new US LNG projects is plausible.

Two factors motivate this scenario. First, the global macroeconomic outlook has become more uncertain amid revived protectionist policies and trade frictions under the Trump administration (2025-29). These include tariffs on construction inputs (e.g., steel and aluminum) and contentious trade negotiations that have dampened growth expectations for major LNG importers such as China and India. In February 2025, China ceased importing US LNG,^76^ raising concerns about the long-term COM viability of new US export terminals and weakening the economic case for additional final investment decisions (FIDs). Additionally, executives of oil and gas majors have, in the autumn of 2025, declared that the expansion of new LNG liquefaction capacity in the US is moving too quickly, risking a market oversupply.^77^

Second, escalating trade tensions have disrupted critical supply chains, raising material costs and delaying construction timelines for several ongoing US LNG projects. Increases in regulatory scrutiny and rising capital costs further compound the challenges project developers face. Collectively, these conditions risk derailing the expansion of US LNG capacity planned between 2026 and 2031.

This scenario assumes that these challenges result in the deferral or cancellation of US LNG export terminals slated to come online in that period, reducing expected global LNG supply by 40–45 bcma in 2027-31. The resulting supply shortfall would tighten global LNG markets and exacerbate Europe’s vulnerability to concurrent shocks, particularly under cold weather conditions and without Russian gas imports.

#### Sensitivity scenarios

In addition to stress-testing the European energy system against extreme weather and global supply disruptions, the scenario framework includes a set of sensitivity cases that reflect broader political and policy uncertainties within the European Union. These scenarios assess how alternative assumptions regarding Russian gas imports and renewable deployment trajectories might affect system resilience and sources of flexibility.

#### Russian import policy sensitivities

The first two sensitivity scenarios explore deviations from the Baseline assumption of a full embargo on Russian gas imports after 2027. While a complete reversal of current policy, such as a return to pre-crisis Russian gas import levels, has been discussed in some policy circles, such a scenario is considered both politically implausible^36^ and strategically irrelevant for long-term planning (see Jasiūnas et al.^31^; Baldursson et al.^22^). For this reason, the analysis adopts a conservative, risk-averse approach aligned with the principle that energy system resilience should not be contingent on re-establishing historically unreliable supply routes.

Nonetheless, political divergence within the EU continues to shape the debate. Several Member States have expressed concern about the blanket ban on Russian gas imports. Hungary and Slovakia have openly opposed sanctions on Russian imports. In contrast, others, such as Austria and Italy, are willing to consider restoring Russian pipeline flows in a post-Ukraine-war context.^78^ The EC’s legislative proposal allows for derogations in cases where national supply security is deemed at risk.^79^ Moreover, legal experts have raised doubts about the adequacy of the proposed “force majeure” provisions to shield European companies from arbitration claims arising from premature contract terminations.^80^

Against this backdrop, the analysis introduces two policy sensitivity scenarios that allow for a limited return of Russian gas imports to Europe beyond 2026 as outlined below. These scenarios allow the model to explore how marginal Russian gas imports could alleviate system stress under tight global LNG conditions and adverse weather events.

1. Russian LNG scenario: Pipeline imports from Russia are banned, but LNG deliveries are permitted up to 30 bcm per year.
2. Russian All scenario: Up to 15 bcm/year of pipeline gas flows via Ukraine, and there is no embargo on Russian LNG.

#### Renewable deployment sensitivity: REPowerEU

The analysis also examines the implications of a more ambitious deployment of renewable energy infrastructure across Europe. As part of its 2050 net-zero commitment, the EU requires each Member State to submit a ten-year National Energy and Climate Plan (NECP), which sets out nationally determined contributions to the collective climate goals. In response to the geopolitical shock of Russia’s invasion of Ukraine, the EC launched the REPowerEU initiative to accelerate the phase-out of Russian fossil fuel imports while reinforcing longer-term decarbonization targets.

Although the 2019 NECP targets are generally considered achievable by Member States, the more aggressive 2030 REPowerEU wind and solar targets are considered highly ambitious, if not unprecedented.^81^ The feasibility of achieving these targets remains uncertain due to permitting bottlenecks, supply chain and investment constraints. Nevertheless, given the central role that REPowerEU plays in the EU’s strategy for energy independence, it is adopted here as a relevant policy benchmark.

The analysis compares the Baseline assumption, where 2030 capacity aligns with NECP 2019 targets, with a sensitivity case in which wind and solar deployment meet REPowerEU levels. Table 6 summarizes the two key policy trajectories.

#### Global gas and electricity market model

A partial market equilibrium model was developed for this paper that requires inputs such as gas supply and electricity generation mix to be taken as given (i.e., the model is not optimising for long-term capacity expansion). Thus, the models require calibration to projections of energy supply mix and demand for the modeling time horizon (Table S1).

The principal objective of this study is to assess the potential impact of a set of climate, technology, and geopolitical scenarios on European and other regional power and gas markets. Thus, the modeling horizon covers 2023–2031 at monthly time steps. In this time frame, it is expected that essential decisions on gas supply and electricity-sector capacity will be made in response to the interplay of risks arising from climate, technology, and geopolitical developments.

Table S2 in the supplemental information, details countries and regions in the gas section of the model. Columns 1 and 3 list gas demand and production nodes, while columns 2 and 4 detail corresponding countries and regions belonging to those nodes. Thus, we have 48 gas-demand nodes and 29 gas-production nodes. Note that the gas demand nodes in Europe are further disaggregated into four: residential (RES), commercial (COM), industrial (IND), and power generation (PWR). Gas and electricity markets are coupled via the power generation nodes. On top of these nodes, the model also has other auxiliary nodes such as cross-border pipeline, LNG transhipment, and gas storage nodes (§A6.4-A.6.6), European industrial demand-side response nodes (§A.6.3), and gas wholesale pricing (“hub”) nodes.

#### Weather scenarios

Explicitly considering possible variations in weather scenarios (so-called climate years, CY) is crucial for gas and electricity markets as both become increasingly dependent on such variability. Energy (gas and electricity) demand and the supply of electricity from variable renewables (wind and solar) and hydro are primarily driven by fluctuations in weather (outside temperature, precipitation, wind speed, and solar radiation), which exhibit significant inter-annual variations (IAV).^49^

Following Ah-Voun et al.,^49^ inter-annual variations in the outside temperature (the primary driver of residential gas and electricity demand) were chosen as critical drivers in constructing the weather scenarios. And so, to be consistent with this set of weather scenarios, the same set of historical years for electricity demand and capacity factors for wind, solar and hydro generation was chosen. A publicly available dataset published by ENTSO-e called “Pan-European Climatic Database (PECD 2021.3)” was used for this.^82^ This comprehensive dataset contains more than 30 climate years of hourly electricity demand, wind and solar capacity factors, and hydro energy inflows for individual electricity bidding zones (more than 140) in Europe.

Unfortunately, some of the parameters we need for the modeling do not cover all climate years in our climate scenarios. The PECD dataset includes capacity factors for wind and rooftop solar PV from 1982 to 2019, and for solar CSP from 1982 to 2018. Hydro energy (water) inflow data covers from 1982 to 2017 in most instances, but for some countries, till 2016. Electricity demand data covers 1982 to 2016. Thus, only the electricity demand and hydro inflow for the mild climate scenario are affected by the PECD limitation for the following countries: DE, ES, FR, GB, GR, LV, NO, and PL. Instead of the missing climate years (2017, 2018, and 2019) in the mild weather scenario, the normal climate scenario was used. Regarding gas security of supply, substituting mild for normal for these countries only slightly overestimated the impact of a gas shortage and only in the electricity market (a normal climate year should have higher electricity demand than in a mild climate year).

#### Electricity market modeling

We explicitly model 29 electricity supply and demand nodes corresponding to 29 countries in Europe (Table S3). Electricity demand projection follows the “National Estimates” (NE) 2025 and 2030 scenario developed as part of the Pan-European Climatic Database (PECD 2021.3) for the 2021 “European Resource Adequacy Assessment” (ERAA) study by ENTSO-e. PECD is also central to the 2022 joint ENTSO-e (electricity) and ENTSO-g (gas) “Ten Year Network Development Plan” (TYNDP) study. The two studies share important common assumptions and scenarios, such as “National Trends” (NT) in TYNDP 2022 and “National Estimates” (NE) in the ERAA study.

The NT and NE scenarios are bottom-up projections for the medium-term, covering 2025 and 2030 (NE/NT). These scenarios depend on detailed and systematic data collection processes by national gas and electricity Transmission System Operators (TSOs), reflecting the latest policy- and market-driven developments discussed at the national level at the end of 2020. This includes, but is not limited to, the National Energy and Climate Plans (NECPs) and additional developments and ambitions (such as national hydrogen strategies). We use NE’s estimate of national electricity demand (annual values are shown in Table S3 for normal CY).

Since PECD only covers electricity demand for two spot years – 2025 and 2030 – we use 2021 historic electricity demand and the projected 2025 demand to linearly interpolate for 2022–2024, and for 2026–2029 we use the annual electricity demand for 2025 and 2030. The year 2031 is assumed to have the same annual electricity demand as in 2030. The procedure for interpolation is as follows.

1. We choose a normal climate year for both 2025 and 2030 to do the interpolation (for results of this interpolation, see Table S3);
2. As a next step to match our calendar years with the chosen climate scenarios, we use the annual interpolated demand values calculated in step 1 and proportionally adjust the corresponding hourly demand time series in the PECD; more specifically:
3. To calculate the hourly demand time series for the years 2022–2024, we use their corresponding annual interpolated values (step 1) and adjust the hourly demand time series in the PECD for the year 2025, ensuring that we choose consistent climate years.
4. Similarly, to calculate the hourly demand time series for 2026–2029, we use their corresponding annual values (step 1) and adjust the hourly demand in the PECD for 2030, considering our mapping between the years and climate scenarios.

The above procedures ensure that we separate the effects of energy policy scenarios on demand from the effects of climate on inter-annual demand variations. That is why we assume a normal climate scenario for all years in the modeling and only then adjust for possible climate impacts on the electricity demand of a particular year within the modeling horizon.

We follow the 2021 ERAA NE scenario, which provides a generation mix for each country for 2025 and 2030. We take the existing generation mix (2021) and the 2025 forecasted mix to derive the generation mix for 2022–2024 (by linear interpolation), and we use 2025 and 2030 to calculate the generation mix for 2026–2029 (by linear interpolation). The 2031 generation mix is assumed to be the same as in 2030.

For Ukraine, we assume the existing generation mix in the modeling. This assumption should not hold if commercial power exchange between Ukraine and European countries is limited. Table S4 shows the generation mix at the EU27+ (Norway, Switzerland, and the UK) level for 2023–2031 using the existing generation mix and the 2025 and 2030 projections to interpolate for the years in between.

#### Gas market modeling

For European countries in the model, we consider the following gas demand sectors.

• Gas demand in residential (RES) buildings is split into gas demand for space heating (temperature dependent) and gas demand for all other purposes (assumed flat across the year);
• Gas demand in commercial (COM) buildings is split into gas demand for space heating (temperature dependent) and gas demand for other purposes (assumed flat);
• Gas demand in industry and road transport (IND); industrial gas demand also includes non-energy use gas demand (i.e., gas as feedstock).
• Gas demand in the electricity generation sector (PWR) is modeled endogenously by coupling gas and electricity markets via gas-fired electricity generation capacity (see §A.4.).

Temperature-sensitive gas demand, especially for household space heating, is the most critical demand category (which falls under the definition of protected customers, especially residential heat demand); we developed a method to model it explicitly.^49^

As for industrial gas demand profiles, we rely on a dataset from Zhou et al.^83^ The dataset contains daily industrial gas demand for EU27+UK for 2016–2022. We use the dataset to create daily profiles by averaging the authors’ simulated daily industrial demand.

Lastly, the energy sector’s own use of gas represents ca. 4.5% of total gas demand, according to Eurostat gas consumption data for 2019. We consider this demand by assuming a 4.5% uplift of the estimated gas consumption in the residential, commercial, and industrial sectors.

For Ukraine, in line with the current electricity consumption trend, we assume that final gas demand will be 40% lower than the pre-war consumption level (2019) for the entire modeled period (2022–2031). Thus, the gas demand for space heating in Ukraine was reduced by 40%, along with final demand. For Norway and Switzerland, we assume no growth in final gas demand.

Industrial demand side response is divided into production curtailment and fuel switching. We discuss production curtailment first, then fuel switching.

We use a detailed dataset of industrial energy consumption structure taken from the IDEES Database^84^ to calculate the cost and capacity of industrial production curtailment. The database reports production capacity, historical output of final products, energy consumption structure and gross value added (GVA) for each of the industrial sectors outlined in the table below. The dataset covers 2000–2015; thus, we calculate the average of these time series. First, we divide the gross value added by natural gas consumption to calculate the unit cost ($/mmBtu of natural gas) of demand response (Equation A1).

(Equation A1) $${\mathrm{Cos}\;t_{IC_{DSR}}}_{i}=\frac{{GVA}_{i}}{Q_{i}}\times \frac{Q_{i}}{C_{i}}$$

where ${GVA}_{i}$ is the gross value added of industry *i*, $Q_{i}$ is the final industrial output, $C_{i}$ is natural gas consumption.

Strictly speaking, the cost of demand response should also include the price of gas at which industrial consumers agreed to pay for the resource. Since our primary objective is to use the computed cost of industrial demand response as an allocation mechanism during a shortage period, the purchase price should not matter, as in Europe, wholesale gas markets are well-integrated. Therefore, only industry- and country-specific GVAs will influence this allocation.

The volume of demand side response from production curtailment is calculated using the capacity utilisation rate (${UR}_{i}$) in 2015, the industry’s installed capacity ($\bar{Q_{i}}$), and gas consumption per unit of output ($\frac{Q_{i}}{C_{i}}$) as follows:

(Equation A2) $${Volume_{IC_{DSR}}}_{i}={UR}_{i}\times \bar{Q_{i}}\times \frac{C_{i}}{Q_{i}}$$

The parameters for utilisation rate and installed capacity were taken from the database for 2015 (the latest available), while gas consumption per unit of output is an average of 2000–2015. In line with the IEA’s findings, we assume that the maximum fuel switching in the industrial sector is seven bcm, which we assume will be triggered at a price above $16/MMBtu (annual average 2022 gas price) (see Ruhnau et al.^68^; Moll et al.^69^; Chiacchio et al.^70^).

We developed a Heating Degree Model (HHD) (for details, see Ah-Voun et al.^49^) to measure the impact of adjusting home thermostats on European residential gas demand (see Figure S1 left panel for estimations and right panel for some measured actual behavioral response to the 2022/23 crisis).

In particular, for every half-degree downward adjustment of home thermostats (relative to reference points, which is national specific; see Ah-Voun et al.^49^), ca. 4.7 bcm of gas demand is saved, with GB, Germany, Italy, France, the Netherlands and Belgium providing a total of 87% of that total response. The Tado data (right panel, Figure S1) suggest that around 5.81 bcm was saved in 2022 due to households’ varying responses using thermostats. IEA^85^ analysis suggests that European households’ behavioral response totaled seven bcm, predominantly by adjusting home thermostats downwards by an average of 0.6°C and some limited fuel-switching.

While some empirical studies have emerged recently (see, e.g., Huebner et al.^86^ study evidencing that British household adjusted their thermostat downwards by one °C during the 2022/23 energy crisis), evidence is still limited on the extent of thermostat adjustment by the whole population in Europe (those with gas boilers) and the extent of fuel switching. Thus, to be consistent with IEA’s empirical findings, we allow for a maximum of seven bcm of the European residential sector. This maximum residential gas demand response equals a downward change in home thermostats between 0.6°C and 0.7°C. Based on growing empirical evidence from the 2022/23 energy crisis (Ruhnau et al.^68^; Moll et al.^69^; Chiacchio et al.^70^; Sperber et al.^87^; Zapata-Webborn et al.^88^), we assume that residential demand response will only be triggered when prices exceed $17.6/MMBtu, which is 10% higher than the assumed trigger price for the industrial fuel switching to reflect that it is costlier for residential consumers to change their behavior. Figure S2 shows an example of the total potential gas demand-side response and associated costs for Germany.

We use the average (2011–2021) actual growth rate in production for countries and regions in the model from the BP Statistical Review of World Energy^1^ and apply this average growth rate to project production profiles until 2031. The outlook for production from the UK is based on National Grid ESO Future Energy Scenarios (2022). The production outlook for the Netherlands includes a policy decision to shut down the Groningen field by 2023. Table S10 summarizes the projected production by regions considered in the modeling.

The projection for LNG export and import capacity addition is based on Eikon’s LNG database of LNG projects. For our projections, we only take the capacity of under-construction plants. Note that no projects are under construction to be delivered beyond 2026. Thus, LNG import capacity in 2027–2031 is set at the level reached by 2026.

### Quantification and statistical analysis

The section below documents a mathematical formulation of the global gas market model. A nomenclature is included in Table S14 in the supplemental information. The objective of this model is to minimise the total cost, consisting of the supply chain’s variable costs for gas and power markets, subject to a set of techno-economic constraints. We use data inputs and assumptions outlined in Supplementary Information 1 to solve this model numerically.

This optimisation problem minimises the total cost of meeting gas and electricity demand, consisting of ((Equation A1a), (Equation A1b), (Equation A1c), (Equation A1d), (Equation A1e), (Equation A1f), (Equation A1g), (Equation A1h), (Equation A1i)) while meeting a set of constraints ((Equation A10), (Equation A11), (Equation A12), (Equation A13), (Equation A14), (Equation A15), (Equation A16), (Equation A17), (Equation A18), (Equation A19), (Equation A2), (Equation A20), (Equation A21), (Equation A22), (Equation A23), (Equation A24), (Equation A25), (Equation A26), (Equation A27), (Equation A28), (Equation A29), (Equation A3), (Equation A30), (Equation A31), (Equation A32), (Equation A33), (Equation A34), (Equation A4), (Equation A5), (Equation A6), (Equation A7), (Equation A8), (Equation A9)).

(Equation A1a) $$\sum_{y,t,z}{gprod}_{y,t,z}\left({gprod}_{y,t,z}\times {gPCOST}_{z,y}^{B}+{gPCOST}_{z,y}^{A}\right)$$

(Equation A1b) $$\sum_{y,t,n,nn}{gflow}_{y,t,n,nn}^{pipe}\times {gFCOST}_{n,nn,y}^{Flow}$$

(Equation A1c) $$\sum_{y,t,n,nn,l}{gflow}_{y,t,n,nn,l}^{LNG}\left({gFCOST}_{l}^{LNG}\times {gSHIPTIME}_{t,n,nn}^{LNG}+{gFCOST}_{n,nn,y}^{Flow}\right)$$

(Equation A1d) $$\sum_{y,t,s}\frac{{gstor}_{y,t,s}^{IN}\left({gSCOST}_{y,s}^{B}\times {gstor}_{y,t,s}^{IN}+{gSCOST}_{y,s}^{A}\right)}{2}+\sum_{y,t,s}\frac{{gstor}_{y,t,s}^{OUT}\left({gSCOST}_{y,s}^{B}\times {gstor}_{y,t,s}^{OUT}+{gSCOST}_{y,s}^{A}\right)}{2}$$

(Equation A1e) $$\sum_{y,t,m}{gloadshed}_{y,t,m}\times {gDCOST}_{y,m}^{LoadShed}$$

(Equation A1f) $$\sum_{y,t,j,i,n}{efueldem}_{y,t,j,i,n}\left({efueldem}_{y,t,j,i,n}\times {eFCOST}_{n,i,y}^{B}+{eFCOST}_{n,i,y}^{A}\right)$$

(Equation A1g) $$\sum_{y,t,j,n}{eoutput}_{y,tj,n}\times {eCI}_{j,n,y}\times {eGCOST}_{n,y}^{Carbon}$$

(Equation A1h) $$\sum_{y,t,j,n}\left({eoutput}_{y,tj,n}+{eEXOG\_GEN}_{y,t,j,n}\right){eGCOST}_{j,n,y}^{Var}$$

(Equation A1i) $$\sum_{y,t,n}{{eloadshed}_{y,t,n}\times eDCOST}_{m}^{LoadShed}$$

where (1a-e) are costs related to gas supply and (1f-i) are costs related to electricity generation.

#### Gas market constraints

(Equation A2) $$\left({gDEM}_{y,t,n}-{gloadshed}_{y,t,n}\right)+{egasdem}_{y,t,n}+{gstor}_{y,t,n}^{IN}+\sum_{nn}{gflow}_{y,t,n,nn}^{pipe}+\sum_{nn,l}{gflow}_{y,t,n,nn,l}^{LNG}=\sum_{nn}{gflow}_{y,t,nn,n}^{pipe}\left(1-{gLoss}_{y,nn,n}^{pipe}\right)+\sum_{nn,l}{gflow}_{y,t,nn,n,l}^{LNG}\left(1-{gLoss}_{y,nn,n}^{LNG}\right)+{gprod}_{y,t,n}+{gstor}_{y,t,n}^{OUT},\forall y,t,n$$

(Equation A3) $${gprod}_{y,t,z}\leq \bar{{gPROD}_{y,z}},\forall y,t,z$$

(Equation A4) $${gflow}_{y,t,n,nn}^{pipe}\leq \bar{{gFLOW}_{y,n,nn}^{pipe}},\forall y,t,n,nn$$

(Equation A5) $$\sum_{l}{gflow}_{y,t,n,nn,l}^{LNG}\leq \bar{{gFLOW}_{y,n,nn}^{LNG}},\forall y,t,n,nn$$

(Equation A6) $$\sum_{n,nn}{gflow}_{y,t,n,nn,l}^{LNG}\times {gDIST}_{n,nn}^{LNG}\leq \bar{{gSHIPSTEP}_{l}^{LNG}}\times \bar{{gSHIP}_{y,t}^{LNG}},\forall y,t,l$$

(Equation A7) $$\sum_{n,nn,l}{gflow}_{y,t,n,nn,l}^{LNG}\leq {gSHIP}^{LNG-Suez},\forall y,t$$

(Equation A8) $$\sum_{n,nn,l}{gflow}_{y,t,n,nn,l}^{LNG}\leq \bar{{gSHIP}^{LNG-PM}},\forall y,t$$

(Equation A9) $$\sum_{n,nn,l}{gflow}_{y,t,n,nn,l}^{LNG}\leq \bar{{gSHIP}^{LNG-NSR}},\forall y,t$$

(Equation A10) $$\sum_{nn}{gflow}_{y,t,n,nn}^{pipe}\leq \sum_{nn}\bar{{gLIQ}_{y,n,nn}^{LNG}},\forall y,t,n$$

(Equation A11) $$\sum_{nn}{gflow}_{y,t,nn,n}^{pipe}\leq \sum_{nn}\bar{{gREGAS}_{y,nn,n}^{LNG}},\forall y,t,n$$

(Equation A12) $${gstor}_{y,t,s}^{IN}\leq \bar{{gSTOR}_{s,y}^{IN}},\forall y,t,s$$

(Equation A13) $${gstor}_{y,t,s}^{OUT}\leq \bar{{gSTOR}_{s,y}^{OUT}},\forall y,t,s$$

(Equation A14) $${gstorlevel}_{y,t,s}^{total}\leq \bar{{gSTOR}_{s,y}^{Level}},\forall y,t,s$$

(Equation A15) $${gstorlevel}_{y,t,s}^{total}\geq \bar{{gSTOR}_{s}^{End}},\forall y,t,s$$

(Equation A16) $${gstorlevel}_{y,t,s}^{total}\geq \bar{{gSTOR}_{y,t,s}^{\mathrm{Min}}},\forall y,t,s$$

(Equation A17) $${gstorlevel}_{y,t,s}^{total}\leq \bar{{gSTOR}_{y,t,s}^{\mathrm{Max}}},\forall y,t,s$$

(Equation A18) $${gstorlevel}_{y,t,s}^{intra}={gstorlevel}_{y,t-1,s}^{intra}+{gstor}_{y,t,s}^{IN}-{gstor}_{y,t,s}^{OUT},\forall y,t,s$$

(Equation A19) $${gstorlevel}_{y,s}^{inter}={gSTOR}_{y,s}^{Init}+{gstorlevel}_{y-1,s}^{inter}+{gstorlevel}_{y-1,t|ord\left(t\right)=card\left(t\right),s}^{intra},\forall y,s$$

(Equation A20) $${gstorlevel}_{y,t,s}^{total}={gstorlevel}_{y,s}^{inter}+{gstorlevel}_{y,t,s}^{intra},\forall y,t,s$$

#### Electricity market constraints

(Equation A21) $$\sum_{j}\left({eoutput}_{y,tj,n}+{eICflow}_{y,tj,n}\right)\times \left(1-{eGEN\_SC}_{j,n}\right)+\sum_{j}\left({eEXOG\_GEN}_{y,t,j,n}-{ecurtail}_{y,t,j,n}\right)+\sum_{j}{estor}_{y,t,j,n}^{OUT}+\sum_{nn}{eflow}_{y,t,nn,n}=\left({eDEM}_{y,t,n}-{eloadshed}_{y,t,n}\right)+\sum_{nn}{eflow}_{y,t,n,nn}+\sum_{j}{estor}_{y,t,j,n}^{IN},\forall y,t,n$$

(Equation A22) $${eoutput}_{y,tj,n}\leq {\bar{eGENCAP}}_{j,n,y},\forall y,t,j,n$$

(Equation A23) $$\sum_{j}{eoutput}_{y,tj,n}\times {eHR}_{j,n,y}\leq {\bar{eFSUPPLY}}_{i,n,y},\forall t,i,n,y$$

(Equation A24) $${estor}_{y,t,j,n}^{IN}\leq \bar{{eSTOR}_{j,n,y}^{IN}},\forall y,t,j,n$$

(Equation A25) $${estor}_{y,t,j,n}^{OUT}\leq \bar{{eSTOR}_{j,n,y}^{OUT}},\forall y,t,j,n$$

(Equation A26) $${estorlevel}_{y,t,j,n}^{total}\leq \bar{{eSTOR}_{j,n,y}^{Level}},\forall y,t,j,n$$

(Equation A27) $${estorlevel}_{y,t,j,n}^{total}\geq \bar{{eSTOR}_{y,j,n}^{\mathrm{Min}}},\forall y,t|ord\left(t\right)=card\left(t\right),j,n$$

(Equation A28) $${eflow}_{y,t,n,nn}\leq \bar{{eFLOW}_{n,nn,y}^{power}},\forall y,n,nn,t$$

(Equation A29) $$-{\bar{eGENCAP}}_{j,n,y}\leq {eICflow}_{y,tj,n}\leq {\bar{eGENCAP}}_{j,n,y},\forall y,t,j,n$$

(Equation A30) $${estorlevel}_{y,t,j,n}^{intra}={estorlevel}_{y,t-1,j,n}^{intra}+\left({eHYDRO}_{y,t,j,n}^{INFLOW}-{ehydrospill}_{y,t,j,n}\right)+{estor}_{y,t,j,n}^{IN}\times \left(1-{eGEN\_SC}_{j,n}\right)-{estor}_{y,t,j,n}^{OUT},\forall y,t,j,n$$

(Equation A31) $${estorlevel}_{y,j,n}^{inter}={eSTOR}_{y,j,n}^{Init}+{estorlevel}_{y-1,j,n}^{inter}+{estorlevel}_{y-1,t|ord\left(t\right)=card\left(t\right),s}^{intra},\forall y,j,n$$

(Equation A32) $${estorlevel}_{y,t,j,n}^{total}={estorlevel}_{y,j,n}^{inter}+{estorlevel}_{y,t,j,n}^{intra},\forall y,t,j,n$$

(Equation A33) $${egasdem}_{y,t,n}=\sum_{j}{eoutput}_{y,tj,n}\times {eHR}_{j,n,y}$$

(Equation A34) $${efueldem}_{y,t,j,i,n}={eoutput}_{y,tj,n}\times {eHR}_{j,n,y}$$

## Supplemental information

Document S1. Figures S1 and S2 and Tables S1–S14

Data S1. Supplemental model outputs
