# Supplementary material for: Stress-testing the EU energy system: Modeling resilience without Russian gas
Source: iScience. 2026 Jun 1;29(6):115866. doi: 10.1016/j.isci.2026.115866 (PMC13253129; doi:10.1016/j.isci.2026.115866)
Supplement: Document S1. Figures S1 and S2 and Tables S1–S14 [file mmc1.pdf]

iScience, Volume *29*

Supplemental information

Stress-testing the EU energy system: Modeling resilience without Russian gas

Chi Kong Chyong and Henrik Schmidt

Figure S. 1: Estimated gas demand-side response using thermostats in residential buildings

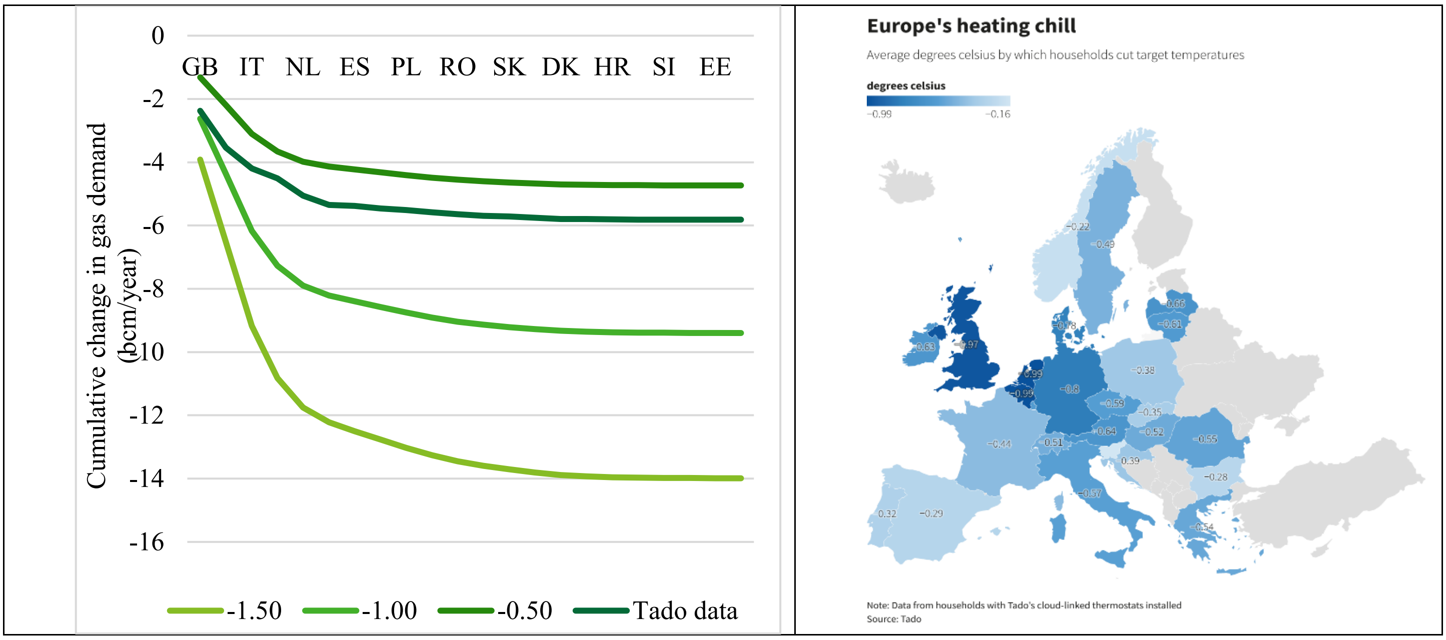

Figure S. 2: An example of the gas demand-side response curve for Germany

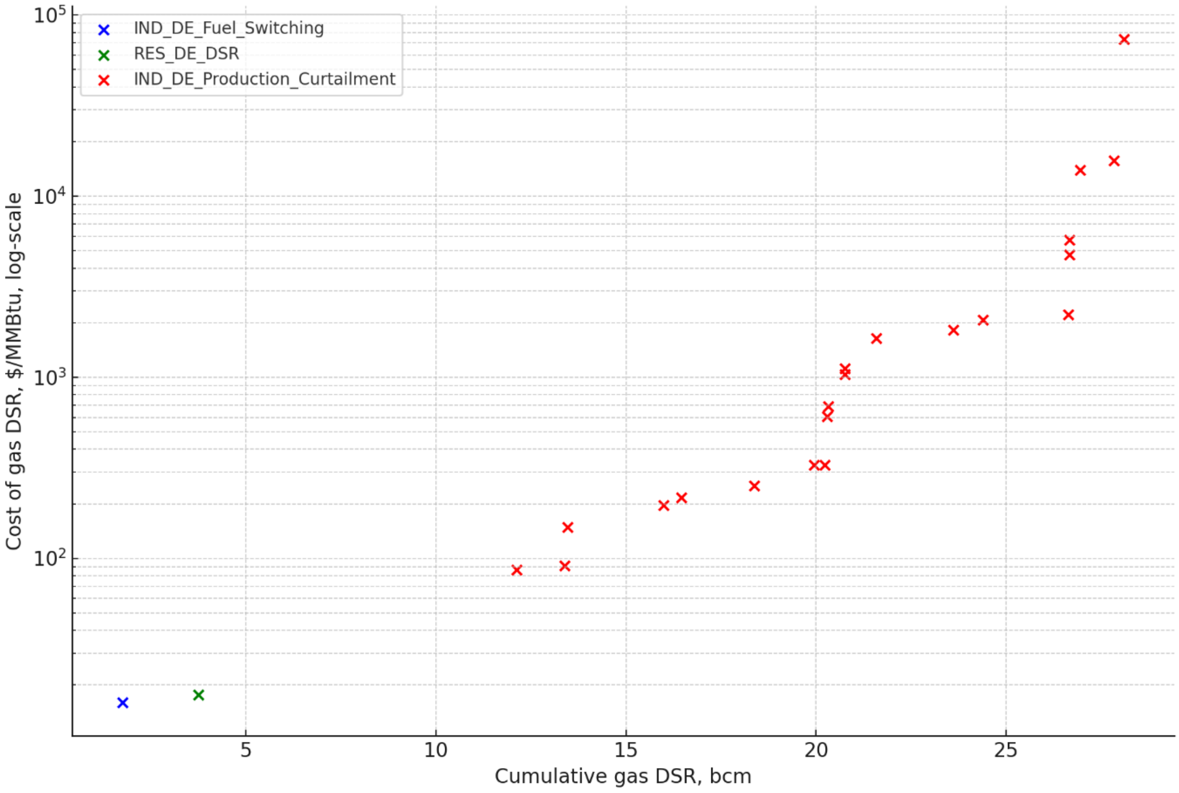

Table S. 1: Summary of input data assumptions and sources

Model inputs	Sources
**Electricity model**	
**Demand**	• Pan-European Climatic Database, Demand dataset for 2025 and 2030; available here.<br>• Climate years were chosen according to climate scenarios developed by Ah-Voun et al. (2024).<br>• For interpolated years (2022-2024), we took the 2021 annual electricity demand from the ENTSOE TP as the starting point and 2025 from the ERAA study.
**Generation mix**	• 2021 ERAA study: National Estimates 2025/2030 Scenario.<br>• PEMMDB National Estimate Excel file; Tab "National Estimates 2025" for generation mix in 2025; Tab "National Estimates 2030" for generation mix 2030.<br>• For interpolated years (2022-2024), we took the existing generation mix from the ENTSOE Transparency Platform (ENTSOE TP). ENTSOE TP does not provide information regarding existing battery capacity, so we use the "Database of the European Energy Storage Technologies and Facilities" to find installed battery capacity. The database is available here.<br>• Techno-economic parameters were taken from PEMMDB (excel file), tab "Thermal Properties."<br>• The thermal efficiency of fossil fuel plants was taken from the JRC Open Power Plants Database v1.00, available here.
**Network**	• 2021 ERAA study: average of top 50 hours of projected Net Transfer Capacity for 2025/2030.<br>• For interpolated years (2022-2024), we took the 2021 highest flow hour from the ENTSOE TP as the starting point and 2025 from the ERAA study.
**Commodity prices**	• 2021 ERAA study and Eikon and Bloomberg terminal (see §A.5.8)
**Natural gas model**	
**Demand**	• Demand projections and climate years were chosen according to climate scenarios developed by Ah-Voun et al. (2024).<br>• IEA reports, Eurostat, and other sources (see §A.6.1-A.6.3)
**Supply**	• BP Statistical Review of World Energy (2022); National Grid ESO Future Energy Scenarios (2022); Chyong and Hobbs (2014); Chyong et al. (2023); JODI Dataset and other sources (see §A6.4)
**Storage**	• IEA (2019) Natural Gas Information Report; the Eikon LNG dataset; EIA's Field Level Storage data for the U.S.A.; ENTSOG 2022 TYNDP and other sources (see §A6.5)
**Transport**	• ENTSO-G; ACER; Chyong and Hobbs (2014); Eikon; GIIGNL and other sources (see §A6.6)

Table S. 2: Gas demand and production nodes in the model

Demand		Production	
Nodes in the model [1]	Countries & regions [2]	Nodes in the model [3]	Countries & regions [4]
Russia	Russia	Algeria	Algeria
Belgium	Belgium	Denmark	Denmark
Germany	Germany	Germany	Germany
France	France	Austria	Austria
South East Asia	Bangladesh	Hungary	Hungary
South East Asia	Brunei Darussalam	Poland	Poland
South East Asia	Indonesia	Romania	Romania
South East Asia	Malaysia	Italy	Italy
South East Asia	Myanmar	Czech Republic	Czech Republic

South East Asia	Philippines	France	France
South East Asia	Singapore	Greece	Greece
South East Asia	Thailand	Slovak Republic	Slovak Republic
South East Asia	Viet Nam	Slovenia	Slovenia
South East Asia	Other Southeast Asia	Bulgaria	Bulgaria
Middle East	Bahrain	Croatia	Croatia
Middle East	Iraq	Spain	Spain
Middle East	Iran	Central Asia	Kazakhstan
Middle East	Jordan	Central Asia	Kyrgyzstan
Middle East	Kuwait	Central Asia	Tajikistan
Middle East	Oman	Central Asia	Turkmenistan
Middle East	Qatar	Central Asia	Uzbekistan
Middle East	Saudi Arabia	South East Asia	Bangladesh
Middle East	Syrian Arab Republic	South East Asia	Myanmar
Middle East	United Arab Emirates	South East Asia	Vietnam
Middle East	Yemen	South East Asia	Malaysia
North America	Canada	South East Asia	Philippines
North America	United States	South East Asia	Thailand
North America	Mexico	South East Asia	Indonesia
Netherlands	Netherlands	South East Asia	Brunei Darussalam
Austria	Austria	South East Asia	Other Southeast Asia
Italy	Italy	Australia	Australia
Switzerland	Switzerland	Trinidad & Peru	Trinidad and Tobago
Slovenia	Slovenia	Trinidad & Peru	Peru
Spain	Spain	Middle East	Bahrain
Portugal	Portugal	Middle East	Iraq
Denmark	Denmark	Middle East	Iran
Poland	Poland	Middle East	Jordan
Czech Republic	Czech Republic	Middle East	Kuwait
Slovak Republic	Slovak Republic	Middle East	Oman
Bulgaria	Bulgaria	Middle East	Saudi Arabia
Romania	Romania	Middle East	Syrian Arab Republic
Latvia	Latvia	Middle East	United Arab Emirates
Hungary	Hungary	Middle East	Yemen
Ukraine	Ukraine	Qatar	Qatar
Turkey	Turkey	Rest of Africa	Angola
Lithuania	Lithuania	Rest of Africa	Cameroon
Greece	Greece	Rest of Africa	Côte d'Ivoire
Moldova	Moldova	Rest of Africa	Egypt
Sweden	Sweden	Rest of Africa	Equatorial Guinea
Croatia	Croatia	Rest of Africa	Gabon
Balkans	Albania	Rest of Africa	Libya
Balkans	Bosnia & Herzegovina	Rest of Africa	Morocco
Balkans	FYROM	Rest of Africa	Mozambique
Balkans	Serbia	Rest of Africa	Nigeria
Great Britain	United Kingdom	Rest of Africa	South Africa
Luxembourg	Luxembourg	Rest of Africa	Tunisia
Rest of Americas	Chile	Rest of Africa	Other Africa
Rest of Americas	Argentina	South Caucasus	Azerbaijan
Rest of Americas	Bolivia	South Caucasus	Georgia
Rest of Americas	Brazil	South Caucasus	Armenia
Rest of Americas	Colombia	Russia	Russia
Rest of Americas	Cuba	Norway	Norway
Rest of Americas	Peru	Netherlands	Netherlands
Rest of Americas	Trinidad and Tobago	North America	Canada
Rest of Americas	Venezuela	North America	United States
Rest of Americas	Other Americas	North America	Mexico
China	Hong Kong, China	UKCS	GB

China	China (People's Rep.)	GB Onshore	GB
India	India	Rest of Americas	Chile
Japan, Korea & Taiwan	Japan	Rest of Americas	Argentina
Japan, Korea & Taiwan	Korea	Rest of Americas	Bolivia
Japan, Korea & Taiwan	Taiwan	Rest of Americas	Brazil
SEM	Ireland	Rest of Americas	Colombia
SEM	Northern Ireland	Rest of Americas	Cuba
SEM	Isle of Man	Rest of Americas	Venezuela
Estonia	Estonia	Rest of Americas	Other Americas
Finland	Finland	China	China
Algeria	Algeria	India	India
Rest of Africa	Angola	Japan, Korea & Taiwan	Japan
Rest of Africa	Congo	Japan, Korea & Taiwan	Korea
Rest of Africa	Côte d'Ivoire	Japan, Korea & Taiwan	Taiwan
Rest of Africa	Egypt	Ireland	Ireland
Rest of Africa	Gabon	Balkans	Albania
Rest of Africa	Libya	Balkans	Bosnia and Herzegovina
Rest of Africa	Morocco	Balkans	FYROM
Rest of Africa	Mozambique	Balkans	Serbia
Rest of Africa	Nigeria	Ukraine	Ukraine
Rest of Africa	South Africa	Pakistan	Pakistan
Rest of Africa	Tanzania	Belarus	Belarus
Rest of Africa	Tunisia	Turkey	Turkey
Rest of Africa	Other Africa	Israel	Israel
Central Asia	Kazakhstan	PNG	Papua New Guinea
Central Asia	Kyrgyzstan	SEM	Ireland
Central Asia	Tajikistan	SEM	Northern Ireland
Central Asia	Turkmenistan	SEM	Isle of Man
Central Asia	Uzbekistan		
South Caucasus	Azerbaijan		
South Caucasus	Georgia		
South Caucasus	Armenia		
Norway	Norway		
Australia	Australia		
Pakistan	Pakistan		
Belarus	Belarus		
Israel	Israel		

Table S. 3: Annual electricity demand projection (TWh) for European countries in the model (normal calendar year)

Country	2023	2024	2025	2026	2027	2028	2029	2030	2031
Austria	68	70	73	75	77	79	80	82	82
Belgium	87	88	89	90	91	93	94	95	95
Bulgaria	36	35	34	34	35	35	36	36	36
Switzerland	63	62	62	62	63	63	63	64	64
Czech Republic	69	71	72	73	73	74	74	75	75
Germany	529	542	554	560	566	572	578	584	584
Denmark	40	42	43	45	47	49	51	53	53
Estonia	9	9	9	9	9	9	9	9	9
Spain	250	254	257	258	259	260	261	261	261
Finland	89	92	94	97	100	102	105	108	108
France	466	466	466	468	470	471	473	475	475
Great Britain	296	292	288	292	297	301	306	310	310
Greece	54	56	57	58	59	60	60	61	61
Croatia	18	18	18	18	18	18	18	18	18
Hungary	46	47	47	48	49	50	51	51	51

Island of Ireland (SEM)	42	44	46	47	48	49	50	51	51
Italy	307	316	324	326	327	328	329	330	330
Lithuania	13	13	14	14	14	14	15	15	15
Luxembourg	6	7	8	8	8	8	9	9	9
Latvia	7	7	8	8	8	8	8	8	8
Netherlands	122	130	138	138	139	139	140	140	140
Norway	145	148	151	154	157	159	162	165	165
Poland	173	172	171	173	175	178	180	182	182
Portugal	50	50	51	52	53	54	55	56	56
Romania	61	62	62	63	63	64	65	65	65
Slovenia	14	15	15	15	15	16	16	16	16
Slovakia	29	29	29	30	30	31	31	31	31
Sweden	143	145	147	148	149	150	151	152	152
Ukraine*	88	88	88	88	88	88	88	88	88

Table S. 4: Generation and storage capacity for EU27, Norway, Switzerland and the UK: ERAA 2021 NE Scenario

	**2023**	**2024**	**2025**	**2026**	**2027**	**2028**	**2029**	**2030**	**2031**
**Generation capacity (GW)**									
Biomass	19	13	7	7	7	7	7	7	7
Nuclear	109	106	103	101	100	99	98	97	97
Offshore Wind	34	40	47	57	68	79	90	101	101
Onshore Wind	209	230	251	263	276	288	300	312	312
Other_RES	18	27	35	36	37	38	38	39	39
Solar_PV	178	205	233	260	288	315	342	369	369
Solar Thermal	4	4	5	6	7	8	8	9	9
Lignite existing	42	38	35	33	30	28	26	24	24
Hard Coal existing	58	46	35	32	30	28	25	23	23
Gas existing	205	189	172	172	171	170	170	169	169
Gas CCGT new	1	2	3	4	4	5	6	7	7
Gas OCGT new	1	1	2	2	2	2	3	3	3
Fossil Oil existing	12	9	6	6	6	6	5	5	5
Hydro Reservoir	66	66	66	66	66	66	66	66	66
Hydro Run of River&Pondage	51	51	51	51	51	51	52	52	52
Hydro PS (open loop)	66	66	66	66	67	68	69	69	69
Hydro_PS (closed_loop)	29	29	29	31	32	34	35	37	37
Battery	9	12	16	22	27	33	38	44	44
**Energy Storage (GWh)**									
Hydro Reservoir	75,990	75,990	75,990	75,992	75,994	75,995	75,997	75,999	75,999
Hydro Run of River&Pondage	84	84	84	84	84	84	84	84	84
Hydro PS (open loop)	108,475	108,475	108,475	108,928	109,381	109,834	110,287	110,740	110,740
Hydro_PS (closed_loop)	544	544	544	549	554	559	564	569	569
Battery	17	24	30	44	58	72	86	100	100

Table S. 5: Fuel ($2021/MWh) and carbon ($2021/tCO$_2$e) price assumptions

	2023	2024	2025	2026	2027	2028	2029	2030	2031
**Nuclear**	2.13	2.13	2.13	2.13	2.13	2.13	2.13	2.13	2.13
**Crude Oil**	49.9	47.3	45.1	43.3	41.9	40.8	40.2	40.1	40.1
**Steam coal***	16	17	16	15	14	13	12	10	10
**CO_2 Price (EU ETS)**	119	119	124	131	136	141	146	146	146

Table S. 6: Supply cost curves for thermal coal

	Slope	Intercept	R^2	Unit of measurement	
				Commodity price	Commodity demand
Steam coal: North America	0.17200	4.92733	0.61	$2021/MWh	TWh/day
Steam coal: Central & South America	4.19314	2.44764	0.75	$2021/MWh	TWh/day
Steam coal: Africa & Middle East	1.18445	6.30152	0.69	$2021/MWh	TWh/day
Steam coal: Russia	0.60077	6.52922	0.36	$2021/MWh	TWh/day
Steam coal: Asia Pacific	0.28916	8.37027	0.66	$2021/MWh	TWh/day

Table S. 7: Projected gas demand (bcm) by sectors in Europe under normal calendar year

	2023	2024	2025	2026	2027	2028	2029	2030	2031
AT_(COM)	0.8	0.8	0.8	0.8	0.8	0.8	0.8	0.8	0.8
AT_(IND)	3.2	3.1	3.0	2.9	2.9	2.8	2.7	2.6	2.7
AT_(RES)	1.9	1.9	1.9	1.9	1.9	1.9	1.9	1.8	1.8
BE_(COM)	1.9	1.8	1.8	1.8	1.8	1.8	1.8	1.8	1.8
BE_(IND)	4.7	4.6	4.5	4.5	4.5	4.5	4.5	4.5	4.6
BE_(RES)	4.1	4.1	4.1	4.1	4.1	4.1	4.1	4.1	4.0
BG_(COM)	0.1	0.1	0.1	0.1	0.1	0.1	0.1	0.1	0.1
BG_(IND)	1.9	2.2	2.4	2.2	2.1	1.9	1.8	1.7	1.7
BG_(RES)	0.1	0.1	0.1	0.1	0.1	0.1	0.1	0.1	0.1
CH_(COM)	0.8	0.8	0.8	0.8	0.8	0.8	0.8	0.8	0.8
CH_(IND)	1.3	1.3	1.3	1.3	1.3	1.3	1.3	1.3	1.3
CH_(RES)	1.4	1.4	1.4	1.4	1.4	1.4	1.4	1.4	1.4
CZ_(COM)	1.2	1.2	1.2	1.2	1.2	1.3	1.3	1.3	1.2
CZ_(IND)	2.7	2.8	2.8	2.8	2.9	3.0	3.0	3.1	3.0
CZ_(RES)	2.0	2.0	2.0	2.0	2.1	2.1	2.1	2.1	2.0
DE_(COM)	9.4	9.3	9.2	9.2	9.2	9.2	9.2	9.3	9.3
DE_(IND)	22.3	21.4	20.7	20.8	21.0	21.1	21.3	21.4	21.9
DE_(RES)	23.8	23.6	23.4	23.4	23.5	23.5	23.5	23.6	23.7
DK_(COM)	0.3	0.3	0.2	0.2	0.2	0.2	0.2	0.2	0.2
DK_(IND)	0.8	0.8	0.7	0.7	0.7	0.7	0.6	0.6	0.6
DK_(RES)	0.7	0.7	0.7	0.7	0.7	0.7	0.7	0.7	0.7
EE_(COM)	0.1	0.1	0.1	0.1	0.1	0.1	0.1	0.1	0.1
EE_(IND)	0.2	0.2	0.2	0.2	0.2	0.2	0.2	0.2	0.2
EE_(RES)	0.1	0.1	0.1	0.1	0.1	0.1	0.1	0.1	0.1
ES_(COM)	1.6	1.6	1.6	1.6	1.6	1.6	1.6	1.6	1.6
ES_(IND)	11.1	11.3	11.5	11.3	11.1	10.8	10.6	10.4	10.2
ES_(RES)	3.3	3.3	3.3	3.3	3.3	3.2	3.2	3.2	3.2
FI_(COM)	0.0	0.0	0.0	0.0	0.0	0.0	0.0	0.0	0.0
FI_(IND)	0.9	0.9	1.0	1.0	1.0	0.9	0.9	0.9	1.0
FI_(RES)	0.0	0.0	0.0	0.0	0.0	0.0	0.0	0.0	0.0
FR_(COM)	6.7	6.7	6.7	6.6	6.6	6.6	6.6	6.6	6.5

FR_(IND)	11.8	11.4	11.1	10.9	10.7	10.6	10.4	10.2	10.9
FR_(RES)	14.4	14.4	14.3	14.2	14.2	14.1	14.1	14.0	14.0
GB_(COM)	7.8	7.8	7.8	7.8	7.8	7.7	7.7	7.7	7.7
GB_(IND)	9.5	9.6	9.7	9.5	9.3	9.1	8.9	8.8	8.8
GB_(RES)	26.0	26.1	26.1	26.0	25.9	25.7	25.6	25.5	25.7
GR_(COM)	0.2	0.2	0.3	0.3	0.3	0.3	0.3	0.3	0.2
GR_(IND)	0.8	0.8	0.9	0.9	0.9	0.9	0.9	0.9	0.9
GR_(RES)	0.6	0.6	0.6	0.6	0.6	0.6	0.6	0.6	0.6
HR_(COM)	0.2	0.2	0.2	0.2	0.2	0.2	0.2	0.2	0.2
HR_(IND)	0.6	0.6	0.7	0.7	0.6	0.6	0.6	0.6	0.6
HR_(RES)	0.5	0.6	0.6	0.6	0.6	0.6	0.5	0.5	0.5
HU_(COM)	1.4	1.4	1.4	1.4	1.4	1.4	1.4	1.4	1.4
HU_(IND)	1.9	1.9	2.0	1.9	1.8	1.8	1.7	1.6	1.7
HU_(RES)	3.6	3.7	3.7	3.6	3.6	3.6	3.5	3.5	3.5
IT_(COM)	8.0	8.0	8.0	8.0	7.9	7.9	7.9	7.9	7.7
IT_(IND)	11.9	11.9	11.9	11.8	11.6	11.5	11.4	11.3	11.6
IT_(RES)	19.9	19.9	19.9	19.8	19.7	19.7	19.6	19.6	19.1
LT_(COM)	0.1	0.1	0.1	0.1	0.1	0.1	0.1	0.1	0.1
LT_(IND)	0.6	0.7	0.8	0.8	0.8	0.8	0.8	0.8	0.8
LT_(RES)	0.2	0.3	0.3	0.3	0.3	0.3	0.3	0.3	0.3
LU_(COM)	0.2	0.2	0.2	0.2	0.2	0.2	0.2	0.2	0.1
LU_(IND)	0.3	0.3	0.3	0.3	0.3	0.3	0.3	0.3	0.3
LU_(RES)	0.3	0.3	0.3	0.3	0.3	0.3	0.3	0.3	0.3
LV_(COM)	0.1	0.1	0.1	0.1	0.1	0.1	0.1	0.1	0.1
LV_(IND)	0.1	0.2	0.2	0.2	0.2	0.2	0.2	0.2	0.2
LV_(RES)	0.1	0.1	0.1	0.1	0.1	0.1	0.1	0.1	0.1
NL_(COM)	3.1	3.1	3.2	3.1	3.1	3.1	3.1	3.1	3.3
NL_(IND)	6.4	6.5	6.7	6.6	6.5	6.4	6.3	6.2	6.4
NL_(RES)	8.3	8.4	8.5	8.4	8.4	8.4	8.3	8.3	8.8
NO_(COM)	0.0	0.0	0.0	0.0	0.0	0.0	0.0	0.0	0.0
NO_(IND)	0.5	0.5	0.5	0.5	0.5	0.5	0.5	0.5	0.5
NO_(RES)	0.0	0.0	0.0	0.0	0.0	0.0	0.0	0.0	0.0
PL_(COM)	1.5	1.5	1.5	1.5	1.5	1.6	1.6	1.6	1.5
PL_(IND)	6.0	6.4	6.8	7.3	7.8	8.3	8.7	9.2	9.5
PL_(RES)	3.6	3.7	3.8	3.9	4.0	4.1	4.3	4.4	4.3
PT_(COM)	0.2	0.2	0.2	0.2	0.2	0.2	0.2	0.2	0.2
PT_(IND)	2.1	2.4	2.6	2.6	2.7	2.7	2.7	2.7	2.8
PT_(RES)	0.3	0.4	0.4	0.4	0.4	0.4	0.4	0.4	0.4
RO_(COM)	0.8	0.8	0.8	0.8	0.8	0.8	0.8	0.8	0.8
RO_(IND)	2.9	2.9	2.9	2.9	2.9	2.9	2.9	2.9	2.9
RO_(RES)	2.6	2.6	2.6	2.6	2.6	2.6	2.6	2.6	2.6
SE_(COM)	0.1	0.1	0.1	0.1	0.1	0.1	0.1	0.1	0.1
SE_(IND)	0.5	0.6	0.6	0.7	0.7	0.8	0.8	0.9	0.9
SE_(RES)	0.0	0.0	0.0	0.0	0.0	0.0	0.0	0.0	0.0
SEM_(COM)	0.6	0.6	0.6	0.6	0.6	0.6	0.6	0.6	0.6
SEM_(IND)	1.7	1.7	1.7	1.7	1.7	1.7	1.7	1.7	1.7
SEM_(RES)	0.8	0.8	0.8	0.8	0.8	0.8	0.8	0.8	0.8
SI_(COM)	0.0	0.0	0.0	0.0	0.0	0.0	0.0	0.0	0.0
SI_(IND)	0.6	0.6	0.6	0.7	0.7	0.7	0.7	0.7	0.7
SI_(RES)	0.1	0.1	0.1	0.1	0.1	0.1	0.1	0.1	0.1
SK_(COM)	0.6	0.6	0.6	0.6	0.6	0.6	0.6	0.6	0.6
SK_(IND)	1.2	1.2	1.3	1.3	1.3	1.3	1.3	1.3	1.3
SK_(RES)	1.3	1.4	1.4	1.4	1.4	1.4	1.4	1.4	1.4
UA_(COM)	0.6	0.6	0.6	0.6	0.6	0.6	0.6	0.6	0.6
UA_(IND)	3.0	3.0	3.0	3.0	3.0	3.0	3.0	3.0	3.0
UA_(RES)	4.8	4.8	4.8	4.8	4.8	4.8	4.8	4.8	4.8

Table S. 8: Projected gas demand (bcm) for non-Europe countries

	2023	2024	2025	2026	2027	2028	2029	2030	2031
Belarus	19.9	19.9	19.9	20.1	20.3	20.5	20.7	20.9	20.9
Moldova	3.3	3.3	3.3	3.3	3.3	3.4	3.4	3.4	3.4
Russia (non-power)*	319.9	318.0	311.5	308.5	305.6	302.1	294.5	291.8	299.0
Australia	42.9	44.1	45.3	46.5	47.7	48.9	50.0	51.1	51.3
Balkans	3.3	3.3	3.3	3.3	3.4	3.4	3.4	3.5	3.5
Central Asia	97.9	97.8	97.8	98.8	99.8	100.7	101.7	102.5	102.5
China (non-power)*	331.8	346.6	357.3	365.5	369.9	377.9	382.0	389.7	392.3
Algeria	49.5	50.2	50.9	51.1	51.3	51.4	51.6	51.7	51.5
Israel	11.0	11.3	11.6	11.6	11.7	11.7	11.7	11.8	11.7
India (non-power)*	53.8	55.9	58.4	65.1	71.2	77.5	84.0	90.0	90.3
Japan, Korea and Taiwan (non-power)*	77.9	78.0	79.7	81.4	83.2	84.9	86.6	88.2	88.1
Middle East (non-power)**	389.3	405.5	420.4	421.0	416.0	411.7	412.2	407.6	405.0
North America (non-power)*	705.0	710.1	710.1	665.4	631.3	597.1	556.1	527.8	520.8
Pakistan	43.6	44.7	45.9	47.2	48.6	49.9	51.3	52.6	52.6
Southeast Asia (non-power)*	108.9	109.7	112.5	116.0	121.6	127.2	130.8	136.2	135.7
South Caucasus	18.3	18.3	18.3	18.5	18.7	18.9	19.0	19.2	20.7
Central and South America (non-power)*	103.8	105.3	106.5	104.9	103.3	103.1	101.6	101.4	101.0
Rest of Africa (non-power)*	63.7	71.5	79.2	79.4	79.6	78.5	78.7	78.9	78.9
Turkey	59.6	59.5	59.5	60.1	60.7	61.3	61.9	62.4	62.4

Table S. 9: Projected electricity generation (TWh) and generation capacity (GW)

		Electricity generation			Electricity generation capacity		
		2019	2025	2030	2019	2025	2030
**China**	Bioenergy	128	229	289	23	41	50
	Coal	4,878	5,179	5,152	1,051	1,132	1,148
	Hydro	1,270	1,297	1,389	356	411	446
	Natural gas	251	402	529	86	120	145
	Nuclear	350	451	648	49	65	93
	Oil	11	7	5	8	8	8
	Other renewables	632	1,325	1,940	416	789	1,147
**India**	Bioenergy	42	67	77	12	13	15
	Coal	1,135	1,206	1,343	235	269	269
	Hydro	175	177	226	49	60	76
	Natural gas	71	94	108	28	30	30
	Nuclear	40	66	109	7	9	16
	Oil	*5*	*7*	*7*	*8*	*8*	*8*
	Other renewables	115	279	590	75	174	345
**Japan**	Bioenergy	52	55	61	9	11	12
	Coal	323	290	239	51	50	41
	Hydro	80	90	92	50	51	51
	Natural gas	346	280	238	84	79	77
	Nuclear	86	120	210	33	34	30
	Oil	35	32	18	35	20	12
	Other renewables	74	110	125	68	100	116
**Russia**	Bioenergy	3	4	9	2	2	3

	Coal	173	150	136	51	42	33
	Hydro	190	196	208	54	56	59
	Natural gas	550	601	601	128	139	138
	Nuclear	200	203	219	30	30	32
	Oil	12	5	4	4	2	2
	Other renewables	2	8	28	1	4	12
**Southeast Asia**	Bioenergy	35	31	45	8	10	12
	Coal	510	588	700	81	106	123
	Hydro	195	180	245	47	56	77
	Natural gas	383	524	581	98	123	142
	Nuclear	-	-	-	-	-	-
	Oil	19	18	18	25	22	21
	Other renewables	41	91	152	18	46	76
**Africa**	Bioenergy	2	5	19	1	2	5
	Coal	259	265	256	50	51	48
	Hydro	141	183	221	36	44	51
	Natural gas	332	345	386	110	123	132
	Nuclear	12	14	28	2	2	4
	Oil	72	71	65	43	35	35
	Other renewables	33	87	219	13	38	94
**Central and South America**	Bioenergy	72	91	98	20	23	24
	Coal	68	43	37	14	14	12
	Hydro	723	801	892	186	195	210
	Natural gas	249	232	254	70	72	84
	Nuclear	23	25	36	4	3	5
	Oil	99	91	79	49	43	37
	Other renewables	100	194	273	34	83	116
**Middle East**	Bioenergy	0	2	7	0	0	1
	Coal	1	11	14	0	3	3
	Hydro	19	24	27	17	17	19
	Natural gas	819	846	1,004	224	267	302
	Nuclear	8	41	49	1	7	9
	Oil	307	310	272	96	95	83
	Other renewables	9	43	107	5	21	50
**North America**	Bioenergy	88	102	111	22	23	25
	Coal	1,152	677	501	266	163	111
	Hydro	688	763	788	196	200	204
	Natural gas	1,922	2,207	2,250	554	593	649
	Nuclear	962	867	812	120	111	104
	Oil	81	34	21	79	47	34
	Other renewables	490	844	1,154	214	364	498

Table S. 10: Projection of gas production capacity (bcm/year) by regions in the model

	**2023**	**2024**	**2025**	**2026**	**2027**	**2028**	**2029**	**2030**	**2031**
Algeria	101.60	104.03	106.51	109.05	111.66	114.32	117.05	119.84	122.70
Denmark	1.08	0.97	0.88	0.80	0.73	0.66	0.60	0.54	0.49
Germany	3.73	3.38	3.06	2.78	2.52	2.28	2.07	1.88	1.70
Austria	0.76	0.69	0.63	0.57	0.52	0.47	0.42	0.38	0.35
Hungary	1.39	1.26	1.15	1.04	0.94	0.85	0.77	0.70	0.64
Poland	3.18	2.88	2.61	2.37	2.15	1.95	1.77	1.60	1.45
Romania	6.96	6.31	5.72	5.18	4.70	4.26	3.86	3.50	3.17

Italy	2.62	2.37	2.15	1.95	1.77	1.60	1.45	1.32	1.19
Czech Republic	0.16	0.15	0.13	0.12	0.11	0.10	0.09	0.08	0.07
France	0.01	0.01	0.01	0.00	0.00	0.00	0.00	0.00	0.00
Greece	0.01	0.01	0.01	0.01	0.01	0.01	0.00	0.00	0.00
Slovakia	0.07	0.06	0.06	0.05	0.05	0.04	0.04	0.03	0.03
Slovenia	0.01	0.01	0.01	0.01	0.01	0.01	0.01	0.01	0.01
Bulgaria	0.06	0.05	0.05	0.04	0.04	0.04	0.03	0.03	0.03
Croatia	0.91	0.82	0.75	0.68	0.61	0.56	0.50	0.46	0.41
Spain	0.06	0.06	0.05	0.05	0.04	0.04	0.04	0.03	0.03
Central Asia	177.96	180.78	183.65	186.56	189.51	192.52	195.57	198.67	201.82
Southeast Asia	252.45	253.00	253.55	254.11	254.66	255.22	255.77	256.33	256.89
Australia	180.11	180.11	180.11	180.11	180.11	180.11	180.11	180.11	180.11
Trinidad and Peru	34.07	33.06	32.07	31.11	30.18	29.28	28.40	27.55	26.73
Middle East*	586.52	612.46	639.56	667.85	697.40	728.25	760.47	794.11	829.25
Qatar	213.95	217.47	221.04	224.67	228.37	232.12	235.94	239.81	243.76
Africa**	165.55	170.13	174.84	179.68	184.65	189.77	195.02	200.42	205.96
South Caucasus	36.50	39.11	41.89	44.88	48.08	51.51	55.18	59.11	63.33
Russia Ural region	619.76	613.44	600.92	598.89	591.03	595.27	606.37	611.53	619.20
Russia Volga region	26.92	25.49	23.22	21.70	19.80	17.88	16.81	15.53	15.71
Russia Siberia region	39.38	57.23	82.34	96.00	116.60	123.93	125.20	129.56	132.34
Russia Far East region	33.94	33.16	32.31	31.79	30.63	30.82	29.48	31.34	30.92
Norway	126.96	126.96	126.96	126.96	126.96	126.96	126.96	126.96	126.96
Netherlands	16.14	13.37	13.12	13.78	13.29	13.23	13.78	13.24	13.36
North America	1,299.31	1,342.25	1,386.60	1,432.43	1,479.76	1,528.66	1,579.18	1,631.36	1,685.27
Great Britain (UKCS)	38.12	34.89	31.93	30.23	27.85	23.91	22.08	19.32	19.32
Great Britain (Onshore)	0.56	0.53	0.50	0.48	0.45	0.43	0.40	0.38	0.38
South and Central America***	118.28	118.85	119.43	120.01	120.59	121.18	121.77	122.36	122.96
China	225.07	240.86	257.77	275.86	295.22	315.95	338.12	361.85	387.25
India	26.30	25.25	24.24	23.27	22.34	21.44	20.58	19.76	18.97
Japan, Korea and Taiwan	2.83	2.83	2.83	2.83	2.83	2.83	2.83	2.83	2.83
Ireland	1.53	1.38	1.24	1.13	1.02	0.94	0.86	0.77	0.77
Balkans	0.47	0.47	0.47	0.47	0.47	0.47	0.47	0.47	0.47

Ukraine	18.40	18.30	18.21	18.12	18.03	17.94	17.85	17.76	17.67
Pakistan	32.17	31.92	31.67	31.42	31.18	30.93	30.69	30.45	30.21
Belarus	0.16	0.16	0.16	0.16	0.16	0.16	0.16	0.16	0.16
Turkey	0.44	0.44	0.44	0.44	0.44	0.44	0.44	0.44	0.44
Israel	10.36	10.36	10.36	10.36	10.36	10.36	10.36	10.36	10.36
Papua New Guinea	14.11	14.11	14.11	14.11	14.11	14.11	14.11	14.11	14.11

Table S. 11: European gas network expansion projects considered in the model

From	To	Project Name	Commissioning Year	Capacity (bcm/year)
Serbia	Bulgaria	Interconnection Bulgaria - Serbia	2022	1.92
Bulgaria	Serbia	Interconnection Bulgaria - Serbia	2022	1.92
Serbia	Bulgaria	Modernisation and rehabilitation of the Bulgarian GTS	2024	0.64
Bulgaria	Serbia	Modernisation and rehabilitation of the Bulgarian GTS	2024	0.64
Germany	Switzerland	TENP Security of Supply plus	2026	2.30
Poland	Ukraine	North-South Gas Corridor in Eastern Poland	2028	3.05
Norway (Dornum)	Denmark	Norwegian tie-in to Danish upstream system	2022	10.08
Denmark	Poland	Poland - Denmark interconnection (Baltic Pipe) - offshore section	2022	10.08
Poland	Denmark	Poland - Denmark interconnection (Baltic Pipe) - offshore section	2022	2.99
Latvia	Lithuania	Enhancement of Latvia-Lithuania interconnection (Lithuania's part)	2023	1.79
Lithuania	Latvia	Enhancement of Latvia-Lithuania interconnection (Lithuania's part)	2023	2.07
TAP Greece	IGB Bulgaria	Interconnector Greece-Bulgaria (IGB Project)	2022	4.66
Greece	IGB Bulgaria	Interconnector Greece-Bulgaria (IGB Project)	2024	3.94
Greece	IGB Bulgaria	Interconnector Greece-Bulgaria (IGB Project)	2027	1.12
Germany	Netherlands	Additional import at Oude StatenZijl area	2022	7.49
Germany	Netherlands	Additional import at Oude StatenZijl area	2025	1.97
Greece	TAP Greece	Booster Compressor Station for TAP in Nea Messimvria	2024	0.99
TAP Greece	Greece	Upgrade of Nea Mesimvria Compressor Station	2023	0.90
IGB Bulgaria	Bulgaria	Interconnector Greece-Bulgaria (IGB Project)	2022	2.96
IGB Bulgaria	Bulgaria	Interconnector Greece-Bulgaria (IGB Project)	2025	1.97

Table S. 12: Projected LNG regasification capacity

		2023	2024	2025	2026	2027	2028	2029	2030	2031
IMPORT CAPACITY	Australia	3	3	3	3	3	3	3	3	3
	Belgium	9	9	9	9	9	9	9	9	9
	China	182	197	205	205	205	205	205	205	205
	Croatia	3	3	3	3	3	3	3	3	3
	Estonia	4	4	4	4	4	4	4	4	4
	Finland	1	1	1	1	1	1	1	1	1

	France	36	36	36	36	36	36	36	36	36
	Great Britain	48	48	48	48	48	48	48	48	48
	Greece	13	13	13	13	13	13	13	13	13
	India	75	75	75	75	75	75	75	75	75
	Israel	5	5	5	5	5	5	5	5	5
	Italy	15	15	15	15	15	15	15	15	15
	Japan, Korea & Taiwan	495	495	495	500	500	500	500	500	500
	Lithuania	4	4	4	4	4	4	4	4	4
	Middle East	65	65	65	65	65	65	65	65	65
	Netherlands	21	21	21	21	21	21	21	21	21
	North America	210	210	210	210	210	210	210	210	210
	Pakistan	13	13	13	13	13	13	13	13	13
	Poland	5	5	5	5	5	5	5	5	5
	Portugal	8	8	8	8	8	8	8	8	8
	Rest of Africa	12	12	12	12	12	12	12	12	12
	Rest of Americas	94	94	94	94	94	94	94	94	94
	South East Asia	86	86	86	86	86	86	86	86	86
	Spain	63	63	63	63	63	63	63	63	63
	Sweden	1	1	1	1	1	1	1	1	1
	Turkey	26	26	26	26	26	26	26	26	26
**EXPORT CAPACITY**	Algeria	34	34	34	34	34	34	34	34	34
	Australia	119	119	119	119	119	119	119	119	119
	Middle East	127	127	172	172	172	172	172	172	172
	North America	116	132	169	204	225	229	229	229	229
	Norway	6	6	6	6	6	6	6	6	6
	Papua New Guinea	11	11	11	11	11	11	11	11	11
	Rest of Africa	70	70	81	81	81	81	81	81	81
	Rest of Americas	27	27	27	27	27	27	27	27	27
	Russia	39	48*	48	48	48	48	48	48	48
	South East Asia	90	90	90	90	90	90	90	90	90

Table S. 13: Additional FSRU terminals in Europe

**Country**	**Start-up**	**Receiving capacity (bcm/y)**	**Storage capacity (bcm)**	**Project/Terminal**
Finland	2022	5.0	0.09	Port of Inkoo
France	2022	4.3	0.09	Le Havre
Germany	2022	7.5	0.10	Brunsbüttel FSRU
Germany	2023	7.5	0.10	Stade FSRU
Germany	2022	7.5	0.10	Wilhelmshaven FSRU
Greece	2024	3.0	0.09	Aegean
Italy	2024	5.0	0.10	Coast of Ravenna
Italy	2023	5.0	0.10	Central- Northern Italy
Latvia	2024	4.1	0.08*	Skulte
Poland	2025	6.0	0.11*	Gdansk

Table S. 14: Nomenclature

	Description	*Units*
**Sets**		
$t \in T$	Discrete time steps representing calendar months considered in the modelling	
$y \in Y$	Discrete time steps representing calendar years considered in the modelling	
$n \in N$	Set of all nodes in the model	
$z \in Z \subset N$	Set of gas production nodes, a subset of all nodes *N*	
$s \in S \subset N$	Set of gas storage nodes, a subset of all nodes *N*	
$m \in M \subset N$	Set of gas market (end-use) nodes, a subset of all nodes *N*	
$l \in L$	Set of steps used to linearise cost functions	
$j \in J$	Set of electricity generation and storage technologies	
$i \in I$	Set of all commodities considered in the model	
**Decision variables - gas**		
$gprod_{y,t,z}$	Gas production at time *t* and year *y*	bcm/m
$gflow^{pipe}_{y,t,n,nn}$	Gas flow via pipelines at time *t* and year *y*	bcm/m
$gflow^{LNG}_{y,t,n,nn,l}$	Gas flow via pipelines at time *t* and year *y*	bcm/m
$gstor^{IN}_{y,t,s}$	Gas storage injection at time *t* and year *y*	bcm/m
$gstor^{OUT}_{y,t,s}$	Gas storage withdrawal at time *t* and year *y*	bcm/m
**Auxiliary variables - gas**		
$gstorlevel^{intra}_{y,t,s}$	Gas storage level at time *t*	bcm
$gstorlevel^{inter}_{y,s}$	Gas storage level at the beginning of year *y*	bcm
$gstorlevel^{total}_{y,t,s}$	Total gas storage level at time *t* and year *y*	bcm
$gloadshed_{y,t,m}$	Gas load shedding at node *m*	bcm/m
**Decision variables - electricity**		
$eoutput_{y,tj,n}$	Electricity generation by technology *j* at *t* and year *y*	MWh_e/m
$eICflow_{y,tj,n}$	Electricity import/export (+/-) with external markets[1]	MWh_e/m
$estor^{IN}_{y,t,j,n}$	Electricity storage injection	MWh_e/m
$estor^{OUT}_{y,t,j,n}$	Electricity storage withdrawal	MWh_e/m
$eflow_{y,t,n,nn}$	Electricity flows between nodes *n* and *nn*	MWh_e/m
$ecurtail_{y,t,j,n}$	Electricity output curtailment	MWh_e/m
$ehydrospill_{y,t,j,n}$	Hydroelectricity output curtailment	MWh_e/m
**Auxiliary variables - electricity**		
$egasdem_{y,t,n}$	Total gas demand for electricity generation	bcm/m
$efueldem_{y,t,j,i,n}$	Fuel demand for electricity generation by generator *j*	MWh_{th}/m
$eloadshed_{y,t,n}$	Electricity load shedding at node *n*	MWh_e/m
$estorlevel^{intra}_{y,t,j,n}$	Electricity storage level at time *t*	MWh_e
$estorlevel^{inter}_{y,j,n}$	Electricity storage level at the beginning of year *y*	MWh_e
$estorlevel^{total}_{y,t,j,n}$	Total Electricity storage level at time *t* and year *y*	MWh_e
**Input parameters - gas**		
$gDEM_{y,t,m}$	Gas demand at node *m*	bcm/m
$\overline{gPROD}_{y,z}$	Gas production capacity	bcm/m
$\overline{gFLOW}^{pipe}_{y,n,nn}$	Gas pipeline flow capacity	bcm/m
$\overline{gFLOW}^{LNG}_{y,n,nn}$	LNG flow capacity	bcm/m
$gLoss^{pipe}_{y,n,nn}$	Gas pipeline losses, as a ratio of gas being sent through *(n,nn)*	Unitless
$gLoss^{LNG}_{y,n,nn}$	LNG shipping losses, as a ratio of LNG being sent through *(n,nn)*	Unitless

$gDIST_{n,nn}^{LNG}$	LNG shipping distance between *n* and *nn*	Nautical miles
$\overline{gSHIP_{y,t}^{LNG}}$	LNG shipping capacity	bcm-nautial miles/m
$gSHIPTIME_{t,n,nn}^{LNG}$	LNG shipping time	days
$\overline{gSHIPSTEP_{l}^{LNG}}$	LNG shipping capacity market segments (to linearise shipping cost function)	Unitless
$\overline{gSHIP^{LNG-Suez}}$	LNG shipping capacity through the Suez Canal	bcm/m
$\overline{gSHIP^{LNG-PM}}$	LNG shipping capacity through the Panama Canal	bcm/m
$\overline{gSHIP^{LNG-NSR}}$	LNG shipping capacity through the North Sea Route	bcm/m
$\overline{gLIQ_{y,n,nn}^{LNG}}$	LNG liquefaction capacity	bcm/m
$\overline{gREGAS_{y,n,nn}^{LNG}}$	LNG regasification capacity	bcm/m
$\overline{gSTOR_{s,y}^{IN}}$	Storage injection capacity	bcm/m
$\overline{gSTOR_{s,y}^{OUT}}$	Storage withdrawal capacity	bcm/m
$\overline{gSTOR_{s,y}^{Level}}$	Storage working volume capacity	bcm
$\overline{gSTOR_{s}^{End}}$	Storage level at the end of the modelling horizon	bcm
$\overline{gSTOR_{y,t,s}^{Min}}$	Storage minimum stock level	bcm
$\overline{gSTOR_{y,t,s}^{Max}}$	Storage maximum stock level	bcm
$gSTOR_{y,s}^{Init}$	Initial storage stock	bcm
$gPCOST_{z,y}^{A}$	Gas production cost function - intercept	\$/tcm
$gPCOST_{z,y}^{B}$	Gas production cost function - slope	\$/tcm^2
$gFCOST_{n,nn,y}^{Flow}$	Gas transport cost	\$/tcm
$gFCOST_{l}^{LNG}$	LNG shipping cost	\$/day/tcm
$gSCOST_{y,s}^{A}$	Gas storage cost function - intercept	\$/tcm
$gSCOST_{y,s}^{B}$	Gas storage cost function - slope	\$/tcm^2
$gDCOST_{y,m}^{LoadShed}$	Gas load shedding cost	\$/tcm
**Input parameters - electricity**		
$eDEM_{y,t,n}$	Electricity demand	MWh_e/m
$eEXOG\_GEN_{y,t,j,n}$	Exogenous electricity generation	MWh_e/m
$eGEN\_SC_{j,n}$	Electricity self-consumption by generator j (a fraction of gross generation)	unitless
$\overline{eGENCAP}_{j,n,y}$	Electricity generation capacity	MW_e
$eHR_{j,n,y}$	Heat rate of generator *j*	MW_{th}/MW_e
$eCI_{j,n,y}$	Carbon intensity of generator *j*	tCO_{2e}/MWh_e
$\overline{eFSUPPLY}_{i,n,y}$	Supply of commodity *i* for power generation	MWh_{th}/m
$\overline{eSTOR_{j,n,y}^{IN}}$	Electricity storage charge capacity	MW_e
$\overline{eSTOR_{j,n,y}^{OUT}}$	Electricity storage discharge capacity	MW_e
$\overline{eSTOR_{j,n,y}^{Level}}$	Electricity storage working volume capacity	MWh_e
$\overline{eSTOR_{y,j,n}^{Min}}$	Electricity storage minimum stock level	MWh_e
$\overline{eFLOW_{n,nn,y}^{power}}$	Electricity cross-zonal flow limit	MWh_e/m
$eHYDRO_{y,t,j,n}^{INFLOW}$	Exogenous hydroelectricity generation	MWh_e/m
$eSTOR_{y,j,n}^{Init}$	Initial electricity storage stock	MWh_e
$eFCOST_{n,i,y}^{A}$	Fuel supply cost function - intercept	\$/MWh_{th}
$eFCOST_{n,i,y}^{B}$	Fuel supply cost function - slope	\$/MWh_{th}^2
$eGCOST_{n,y}^{Carbon}$	Fuel supply cost function - intercept	\$/tCO_{2e}

$eGCOST_{j,n,y}^{Var}$	Variable generation cost	\$/MWh$_e$
$eDCOST_m^{LoadShed}$	Electricity load-shedding cost	\$/MWh$_e$
